# Effects of Binding between Ca in Hard Water and Phosphorus in Amylopectin on the Qualities of Boiled Rice and Rice Noodle Prepared by Soaking and Boiling in Hard Water

**DOI:** 10.3390/foods13132094

**Published:** 2024-07-01

**Authors:** Sumiko Nakamura, Junji Katsura, Akira Suda, Yasuhiro Maruyama, Ken’ichi Ohtsubo

**Affiliations:** 1 Faculty of Applied Life Sciences, Niigata University of Pharmacy and Applied Life Sciences, 265-1, Higashijima, Akiha-ku, Niigata 956-8603, Japan; snaka@nupals.ac.jp; 2 NSP Ltd., Nakanoki 2-31-5-B, Funabashi-shi, Chiba 274-0826, Japan; jkatsura@nsp-jpn.com (J.K.); akira-suda@nsp-jpn.com (A.S.); yasuhiro-maruyama@nsp-jpn.com (Y.M.)

**Keywords:** amylopectin, calcium, hard water, phosphorus, weakly acidic hard water

## Abstract

Recently, global warming has led to an increase in chalky rice grains. This has consequently resulted in the deterioration in quality of rice products. Although we previously reported that hard water, rich in Ca, is useful for the quality improvement of high-temperature-damaged rice grains, the mechanism was not elucidated sufficiently. Therefore, we used various kinds of rice cultivars, from waxy to high-amylose ones, for soaking and boiling in hard water and compared physical and chemical properties of the products. It was shown that the degree of quality improvement, such as final viscosities in pasting property, and textural properties of boiled rice, was more remarkable for high-amylose rice than low-amylose rice. As we found that the phosphorus contents showed positive correlations with amylose and long chains of amylopectin, we estimate that the effects are mainly due to binding of calcium and phosphorus. Because that high-amylose or long-chain-rich amylopectin rice cultivars showed high calcium contents in rice products, these rice cultivars would be very useful to supply calcium through dietary intake via hard water cooking.

## 1. Introduction

Rice (*Oryza sativa* L.) is one of the most important staple crops, feeding almost half of the global population. It is widely cultivated around the world and mainly in Asia [1].

It is well known that calcium is an essential mineral for humans; nevertheless, dietary calcium intake remains insufficient among Asian populations [2,3]. Osteoporosis is now acknowledged as the most prevalent bone disorder in the world [4]. Furthermore, during magnesium depletion, intracellular calcium rises, therefore calcium plays an important role in skeletal and smooth muscle contraction [5]. With the constant increase in the aging population, not only in developed countries but also in South America, Asia, and Africa, osteoporosis is becoming an increasingly worldwide major public health problem [6].

An IPCC special report presented the impacts of global warming of 1.5 °C above pre-industrial levels and related global greenhouse gas emission pathways [7,8,9,10]. Global warming has led to an increase in chalky grains of rice, which has also caused the deteriorations of physicochemical and cooking qualities of rice grains [11,12]. These chalky rice grains are characterized by high α-amylase activity [13], high protease activity, xylanase activity and low apparent amylose contents [14], low cellulase activity, low degree of hardness and stickiness of boiled rice grains than those of the whole rice grains [15,16].

In order to prevent the above-mentioned deterioration in the texture of boiled rice grains from chalky rice, we tried to use hard water for soaking and boiling. It was shown that hard water is useful for the prevention of texture deterioration of the boiled rice grains due to the inhibition of endogenous hydrolytic enzymes, such as amylase, proteinase, and xylanase [16]. Furthermore, we found that hard water is useful for increasing dietary calcium and magnesium intake with the boiled rice grains soaked and cooked using hard water [16]. Nevertheless, elucidation of the mechanism behind improvements in the texture of boiled rice grains has not been established yet.

Unpolished rice grains contain nutritional components, such as carbohydrates (74.0%), proteins (7.4%), fats (3.0%), minerals (1.2%), and dietary fibers (3.0%) [17], and phytic acids, flavonoids, tocopherols, γ-oryzanol, and E and B vitamins are 2–10 times higher in unpolished rice than in polished rice grains [18].

Kubo and Saio reported that the phosphorus content of unpolished rice showed a positive correlation with ripening period and temperature [19]. In rice grains, about 80% of the total phosphorus is found in the form of phytic acid [20]. Ogawa et al. reported that the accumulation site of phytic acid in rice and wheat grains is in the outer region, aleurone layer [21]. Tabata et al. showed that phosphorus in cereal starches exists as two kinds of phosphorus types, one is mainly bound to C_6_ of glucose residue of amylopectin and the other is in the form of amylose–lipid complexes [22].

Wariyah et al. investigated the binding of calcium and rice in the Ca-fortified rice using infra-red analysis, and they concluded that calcium fixation in rice can occur through the formation of hydrogen bonds between starch and Ca-hydrated ionic-dipole bonds [23]. Noda et al. reported that calcium-fortified potato starch was prepared by immersion in natural mineral hard water, and their pasting properties were improved in terms of stability in viscosity [24,25].

Shibuya et al. [26,27] showed that the endogenous xylanase and cellulase play important roles in determining the texture of cooked rice grains similarly with amylose content. In a previous paper, we found high-homology in the DNA sequences encoding 1, 4-β-xylanase and endo-1, 4-β-glucanase for *Indica* rice, *Indica-Japonica* hybrid rice, and *Javanica* rice [28]. Tsujii et al. have reported that hardness of boiled rice showed a negative correlation with extent of decomposition of pectin [29]. Minerals play important roles in the cell walls of plants. The binding of calcium (Ca) and boron (B) to homogalacturonan and RG-II domains of pectin, affect physical and chemical properties of the cell wall through formation of pectic network in primary cell walls [30].

As we described before, physical properties of boiled rice grains are influenced by the characteristics of various rice starches and high-temperature ripening [15,16]. For this reason, it seems that investigating the effects of high-temperature ripening on the various rice cultivars with wide-range starch structures and properties is very important. Specifically, investigations concerning the relationship between physicochemical properties of boiled rice and phosphoric group in various starch from wide-range rice cultivars seems to be a meaningful research subject [16,19].

In our previous paper, we reported that *ae* mutant rice grains lack the starch branching enzyme (BE)IIb, which accelerates the branching of starch molecules. Therefore, *ae* mutant rice shows low amounts of short-chain glucans and high apparent amylose contents (AAC) [31]. In addition to amylose, amylopectin also greatly affects the gelatinization properties of rice starch and the quality of boiled rice grains [32]. Starch microstructures of various kinds of rice cultivars are very diversified, e.g., waxy, low-amylose, high-amylose, *ae*-mutant high amylopectin long chain, etc., [33]. Focusing on the relationship between phosphorus, which increases under high-temperature ripening, and various kinds of starch microstructures, investigations seeking the elucidation of the mechanism by which calcium in hard water is effective to improve the physical properties of high-temperature damaged rice grains [16] would be indispensable and very meaningful.

And the development of high-calcium functional rice products for prevention of calcium depletion would be very meaningful as a counter measure in addition to the development of curing technology for osteoporosis.

Therefore, it would be promising to utilize hard water to improve both the physicochemical and bio-functional qualities of various processed rice products, such as boiled rice, rice crackers, and rice noodles, etc.

## 2. Materials and Methods

### 2.1. Materials

The unpolished rice samples, harvested in 2022 (*Japonica* subspecies), were purchased in 2023 at a local market and were subjected to the measurement of phosphorus contents (*n* = 32). The ordinary *Japonica* rice included *Akitakomachi* (Ibaraki prefecture), *Akitakomachi* (Chiba), *Akitakomachi* (Akita A), *Akitakomachi* (Akita B), *Tsuyahime* (Shimane), *Tsuyahime* (Yamagata A), *Tsuyahime* (Yamagata B), *Tsuyahime* (Miyagi), *Haenuki* (Yamagata), *Sasanishiki* (Miyagi), *Yuudai 21* (Tochigi), *Ginnoshizuku* (Iwate), *Kazesayaka* (Nagano), *Tsugaruroman* (Aomori), *Hatsushimo* (Gifu), *Aichinokaori* (Aichi), and *Gohyakukawa* (Yamanashi) (*n* = 17). The high-quality premium *Japonica* rice included *Koshihikari* (Saga), *Koshihikari* (Ibaraki A), *Koshihikari* (Ibaraki B), *Koshihikari* (Shimane), *Koshihikari* (Niigata A), *Koshihikari* (Niigata B), *Koshihikari* (Niigata C), *Koshihikari* (Yamagata A), *Koshihikari* (Yamagata B), *Koshihikari* (Ishikawa A), and *Koshihikari* (Ishikawa B) (*n* = 11). Low-amylose *Japonica* rice included *Milky queen* (Yamagata), *Milky queen* (Kyoto), *Yumepirika* (Hokkaido A), and *Yumepirika* (Hokkaido B) (*n* = 4) as shown in Table 1.

Moreover, the polished rice samples, harvested in 2022 (various rice subspecies, *n* = 7), were subjected to the measurement of pasting properties, textural properties and mineral contents of boiled rice, and mineral contents of rice noodles and rice crackers. Seven rice cultivars were as follows; Glutinous rice was *Koganemochi* (Niigata). Ordinary *Japonica* rice was *Kinuhikari* (Hyogo). High-quality premium *Japonica* rice was *Koshihikari* (Niigata). Low-amylose *Japonica* rice was *Milky-queen* (Niigata). High-amylose *Japonica* rice was *Koshinokaori* (Niigata). *Japonica-Indica* hybrid rice was Hoshiyutaka (Niigata). *Ae* mutant *Japonica* rice was *Niigata 129gou* (Niigata) as shown in Table 1.

And ordinary *Japonica* rice, Tennotsubu (Fukushima) was used for sensory evaluation test.

Commercial hard waters, Evian (hardness: 304 mg/L, pH: 7.2) and Contrex (hardness: 1468 mg/L, pH: 7.2), were purchased at a local market in Niigata city and used for boiling.

### 2.2. Measurement of the Moisture Content of Rice Flour

The moisture contents of the polished or unpolished rice flours were measured using an oven-drying method by drying 2 g flour samples for 1 h at 135 °C [34].

### 2.3. Preparation of Milled Rice Flour

Polished rice flour was prepared using a cyclone mill (SFC-S1; UDY, Corp., Fort Collins, CO, USA) with a screen containing 1-mm-diameter pores.

### 2.4. Preparation of Starch Granules

Starch granules were prepared from 7 various types of rice flour using the cold alkaline method [35].

### 2.5. Iodine Absorption Spectrum

The iodine absorption spectra of 7 alkali-treated various rice starches were measured using a Shimadzu UV-1800 spectrophotometer. The AACs of alkali-treated rice starch were measured using the iodine colorimetric method of Juliano [33,36]. The absorbance was measured at 620 nm (following Juliano’s method), λ_max_ (peak wavelength on iodine staining of starch, which shows high correlation with the length of glucan chain; molecular size of amylose and super-long chain (SLC)), and absorbance at λ_max_ (A_λmax_).

A degree of polymerization higher than 37% (Fb_3_) was estimated using the following Equation (1) [33].

Fb_3_ (DP ≧ 37) % = 44.691 × *A_λmax_* − 0.774 (1)

### 2.6. Pasting Properties

The pasting properties of starches from 7 various rice samples were measured using a Rapid Visco Analyzer (RVA) (model Super 4 and novel high-pressure-type RVA4800; Newport Scientific Pty Ltd., Warriewood, Australia). Each 3.5 g starch sample was added to 25 mL of distilled water in aluminum cup, respectively. A programmed heating and cooling cycle was as follows: 1.0 min of heating at 50 °C, 6.5 min of heating from 50 to 120 °C, maintenance for 2.0 min at 120 °C, 6.5 min of cooling from 120 °C to 50 °C, and 3.0 min at 50 °C [37]. Thereafter, we measured pasting properties of the polished rice flours using the condition reported by Toyoshima et al.; 1 min of heating at 50 °C, 4.0 min of heating from 50 to 93 °C, maintenance for 7.0 min at 93 °C, 4.0 min of cooling from93 °C to 50 °C, and 3.0 min at 50 °C [38].

Novel indices such as the ratio of setback to consistency (Set/Cons) (positive indices of proportion of amylopectin (DP ≧ 13)) and the ratio of maximum viscosity to final viscosity (Max/Fin) (negative indices of proportion of amylopectin (DP ≧ 13)) were reported to be correlated very strongly with the proportion of intermediate and long chains of amylopectin: Fb_1+2+3_ (DP ≧ 13) [39].

### 2.7. Measurements of Textural Properties of Boiled Rice Grains

The 10 g of the polished rice was added to 16 g (1.6 times, *w*/*w*) of purified water in an aluminum cup as control samples, and another set of rice grains samples (10 g) were added to 16 g (1.6 times, *w*/*w*) of Contrex or weakly acidic Contrex (pH 4.6) [16]. The physical properties of boiled rice grains were measured based on bulk measurements (10 g), which included a low compression (compression ratio = 23%: twice), an intermediate compression (compression ratio = 46%: twice), and a high compression (compression ratio = 92%: twice) according to the 2 × 3 bite method using a Tensipresser (My Boy System, Taketomo Electric Co., Tokyo, Japan) according to the method described by Okadome et al. [40]. For analysis of calcium and magnesium contents, boiled rice was prepared by “Yutori method” reported by Chikubu et al. [41]. The “Yutori method” is a typical rice boiling method usually used in southeastern Asia. Boiling rice in an excess amount of water, followed by discarding water, and simmering by weak heating. In our experiment, 8 g of the polished rice was added in wire mesh basket hanging to tall beaker, which was added to 160 g (large excess of water) of purified water or Contrex, and soaked for 30 min at 25 °C in water bath, respectively. These samples were boiled in an electric rice cooker (SR-SW 182 National, Japan) for 20 min. Immediately after, the samples were drained completely of water. These boiled rice samples were prepared for analysis by pulverizing after lyophilization. 

### 2.8. Xylanase Activity

Xylanase activity of various polished rice flours (*n* = 7) was determined using a kit (Megazyme International Ireland Ltd., Wicklow, Ireland) according to the method described by Nakamura et al. [16].

### 2.9. Measurement of Color Difference of Gelatinized Paste and Boiled Rice Soaked and Boiled in 2 Types of Hard Water or Weakly Acid Hard Water Using Koshihikari as Polished Rice

The color differences of boiled rice grains prepared by soaking and boiling in 2 kinds of hard water (Evian; pH 7.2), (Contrex; pH 7.2), 2 kinds of weakly acidic hard water (Evian; pH 4.6), (Contrex; pH 4.6), or purified water were measured using a color difference meter (Color Meter NW-11, Nippon Denshoku Co., Tokyo, Japan), and also color of rice flour paste, which had been prepared, in 2 kinds of hard water (Evian; pH 7.2), (Contrex; pH 7.2), 2 kinds of weakly acidic hard water (Evian; pH 4.6), (Contrex; pH 4.6) or purified water, during the pasting property test by an RVA, was measured.

### 2.10. Preparation of Rice Noodles

Based on the preparation method for rice noodles [42], 7 kinds of rice flour samples (150 g, each) were added with 90 g of purified water, Contrex (pH 7.2) or Contrex (pH 4.6) at 90 °C, followed by kneading for 20 min with hands, respectively. Thereafter, the dough was stood overnight in a refrigerator. The dough was then put through the roll (100 mm in width and 2 mm of clearance) twice and finally cut by the blade to a width of 2.2 mm. The noodles were heated for 2 min in boiling water and then cooled for 1 min in water at 20 °C [42]. These noodle flour samples were prepared by pulverizing after lyophilization.

### 2.11. Preparation of Rice Crackers

Based on the preparation method for rice crackers, polished rice flour sample (150 g) of Milky Queen and 20 g of potato starch was added with 90 g purified water, Contrex (pH 7.2) or Contrex (pH 4.6) at 90 °C, followed by kneading for 20 min with hands, respectively. Thereafter, the dough was stood overnight in a refrigerator. The dough was cut by the blade to a thickness of 3.0 mm. The rice crackers were prepared by baking at 220 °C for 10 min in steam microwave oven. These rice crackers samples were prepared by pulverizing after lyophilization [43].

### 2.12. Analysis of Calcium, Magnesium and Phosphorus Contents

The calcium and magnesium contents of the boiled rice grains, rice noodles or rice crackers soaked and boiled in Contrex (pH 7.2), weakly acidified Contrex (pH 4.6), or purified water were analyzed by an ICP (Inductively Coupled Plasma) emission spectrometry [44]. The absorbance was measured at 423 nm for calcium, and that of magnesium was 285 nm. And the phosphorus contents of various unpolished rice or polished rice were analyzed by molybdenum blue method [45]. The absorbance was measured at 823 nm. Measurements of the microminerals of rice samples were carried out by the Japan Food Research Laboratories or General Incorporated Association Kenou Research Laboratories.

### 2.13. Sensory Evaluation of Boiled Rice or Rice Noodles after Soaking in Hard Water or Weakly Acidic Hard Water

Sensory test of boiled rice or rice noodles soaked and boiled in different types of water was conducted by 10 trained taste panelists, and the attributes of sensory evaluation were appearance, aroma, hardness, stickiness, taste, and overall evaluation. “Tennotsubu” of polished rice (Fukushima prefecture) was used as boiled rice and “Milky queen” (Niigata prefecture) was used for rice noodle.

### 2.14. Statistical Analyses

We used Excel Statics (ver. 2006; Microsoft Corp., Tokyo, Japan) for the statistical analysis of the significance of regression coefficients using Student’s *t*-test, one-way analysis of variance, and Tukey’s test. And the method of Tukey’s multiple comparison was statistically analyzed using Excel NAG Statistics add in 2.0 (The Numerical Algorithms Group Ltd., Tokyo, Japan).

## 3. Results and Discussion

### 3.1. Phosphorus Contents of 32 Unpolished Rice Samples

**Figure 1:**
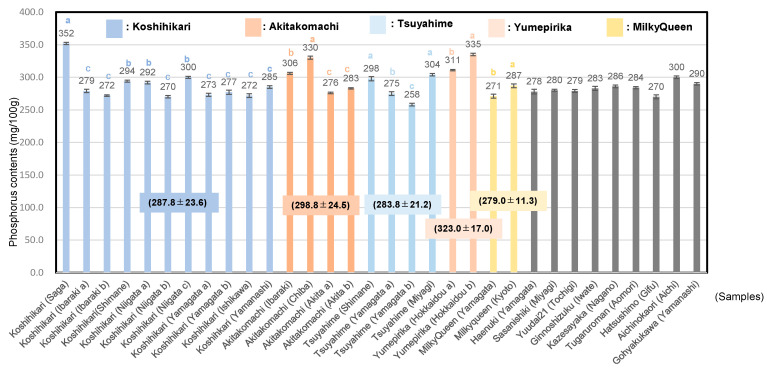
Phosphorus contents of 32 *Japonica* unpolished rice samples. Values are shown as mean ± standard deviation. For phosphorus contents among the same rice cultivars, different letters (a, b, c) indicate significant difference. Correlation is significant at 1% by the method of Tukey’s multiple comparison.

Most starches of cereals, roots, tubers, and legumes, contain 0.02–0.06% of phosphorus in the form of phospholipid, whereas waxy starches have much less phosphorus (0.01% or less) in the form of starch phosphate monoesters [46]. Phosphate monoesters of the starch are located more on the primary carbon (C-6) than on the secondary carbon (C-3) of the anhydrous glucose unit. Sposito showed that activity of inorganic components in the soil solution showed significant positive correlation with the amount of mineral uptake by plants [47]. Suzuki et al. showed that potassium, magnesium and phosphorus contents of 8 pigmented rice were higher than ordinary cultivar throughout 4 years [48,49]. Zeng et al. analysed mineral element contents of the core collection of Yunnan rice, 0and reported that P, K, Ca, and Mg contents were closely correlated to the most plant morphological and grain quality traits [50,51]. Yamakawa et al. showed that high temperature increased sucrose and amino acids and decreased levels of sugar phosphates and organic acid in rice grains [52]. Sugar phosphates are not only involved in metabolic regulation and signaling but also involved in the synthesis of other phosphate compounds [53]. As shown in Figure 1, the phosphorus contents of 32 *Japonica* unpolished rice samples in 2022 were 289.1 ± 20.5 mg/100 g (*n* = 32); those of Koshihikari were 287.8 ± 23.6 mg/100 g (*n* = 11) and those of Akitakomachi 298.8 ± 24.5 mg/100 g (*n* = 4), Tsuyahime 283.8 ± 21.2 mg/100 g (*n* = 4), the other cultivars 283.3 ± 8.4 mg/100 g (*n* = 9), Yumepirika 323.0 ± 17.0 mg/100 g (*n* = 2) and Milky Queen 279.0 ± 11.3 mg/100 g (*n* = 2). Particularly, Koshihikari from Saga prefecture showed the highest phosphorus content.

We investigated correlation between phosphorus contents and sunlight hours among 32 *Japonica* unpolished rice samples [54], as a result, those phosphorus contents showed significant correlation with sunlight hours at the level of 1% as shown in Supplementary Figure S1. As a result, it seems that relationship between ripening temperature and phosphorus content of unpolished rice are similar to that reported by Kubo and Saio’s paper [19]. Although the inorganic components of unpolished rice have a little association with the amount of N, P, K applied as fertilizer, phosphorus contents showed stronger positive correlation with ripening period and temperature rather than with fertilizer [19]. Yamaji et al. developed a genetically modified knock-out rice, of which total phosphorus and phytate contents in the unpolished rice were 20–30% lower than the ordinary lines, whereas yield, seed germination and seedling vigour were not affected [55]. A reduction in phoshorus accumulation in the grain would contribute to sustainable and environmentally friendly agriculture to field and waterways by inhibiting eutrophication of phosphorus in global warming [55].

In this paper, we evaluated the relationship between the phosphorus contents and starch molecular structure. Firstly, as shown in Table 2, among phosphorus contents of 7 various polished rice samples, Niigata 129gou (*ae* mutant *Japonica* rice) and Koshinokaori (high-amylose *Japonica* rice) showed higher values, and Koganemochi (glutinous rice), Kinuhikari (ordinary *Japonica* rice), Koshihikari (high-quality premium *Japonica* rice), Hoshiyutaka (*Japonica-Indica* hybrid rice), and Milky-Queen (low-amylose *Japonica* rice) showed intermediate values.

### 3.2. Iodine Absorption Spectrum for the Survey of Starch Microstructure

In this paper, we evaluated the relationship between the phosphorus contents and wide-range starch molecular structures.

We investigated the starch molecular structure of 7 kinds of rice samples, including *ae* mutant *Japonica* rice (Niigata 129gou), high-amylose *Japonica* rice (Koshinokaori), glutinous *Japonica* rice (Koganemochi), ordinary *Japonica* rice (Kinuhikari), high-quality premium *Japonica* rice (Koshihikari), *Japonica-Indica* hybrid rice (Hoshiyutaka) and low-amylose *Japonica* rice (Milky Queen), according to the iodine colorimetric scanning method reported in our previous reports [10,38].

As shown in Table 2, AAC of Niigata 129gou was very high, and those of Koshinokaori and Hoshiyutaka were slightly higher, whereas those of Koshihikari and Kinuhikari were intermediate, and that of Milky Queen was low, and that of Koganemochi was the lowest.

*Ae* mutant showed very high AAC. The *ae* mutation caused a dramatic reduction in the activity of BEIIb, and the activity of soluble starch synthase SSIin the ae mutation was significantly lower than the wild type [56]. As the amylopectin of ae mutants has more long-chain glucans, it tends to take a helical structure in solution [31]. It is known that the cavities in helixes easily make inclusion complexes with low molecular weight substances [57]. The AAC contents are higher than the actual amylose contents because not only amylose but also long-chain amylopectin bind with iodine [58,59,60].

The difference of λ_max_ values tends to reflect amylose molecular sizes (the length of the glucan chain; molecular size of amylose and SLC) [61].

As shown in Table 2, λ_max_ values of Koshinokaori and Hoshiyutaka were very high, and that of Niigata 129gou was slightly higher, whereas those of Koshihikari and Kinuhikari were intermediate, and Milky Queen showed very low value, furthermore Koganemochi showed the lowest value.

The Aλ_max_ values reflects not only the properties of amylose but also those of the amylopectin chain length [61].

As shown in Table 2, Aλ_max_ value of Niigata 129gou was very high, and those of Koshinokaori and Hoshiyutaka were high, whereas Koshihikari and Kinuhikari showed intermediate values, moreover Milky Queen showed very low value and Koganemochi showed the lowest value. Niigata 129gou belongs to ae mutation rice cultivar, that is, 1.4 times greater than that of the high-amylose rice, Koshinokaori; 1.1 times higher than that in the *Japonica-Indica* hybrid rice, Hoshiyutaka; 1.3~1.5 times higher than those of the high-quality premium *Japonica* rice and ordinary *Japonica* rice, Koshihikari and Kinuhikari.

In the previous study, we showed that theλ_max_/Aλ_max_ ratios were negatively correlated with AAC [33]. As shown in Table 2, λ_max_/Aλ_max_ ratio of Milky Queen was very high, and those of Kinuhikari and Koganemochi were high, whereas Koshihikari showed an intermediate value, and Hoshiyutaka and Koshinokaori showed low values, and Niigata129gou showed very low value.

In the previous study, we developed the novel estimation formulae for the ratio of amylopectin chain lengths Fb_3_ (degree of polymerization, DP ≧ 37) % on the basis of the iodine absorption curve [33]. As shown in Table 3, Fb_3_ of Niigata 129gou was very high, and those of Koshinokaori and Hoshiyutaka were slightly higher, whereas Koshihikari and Kinuhikari showed intermediate values, and Koganemochi showed lower value, moreover Milky Queen showed the lowest value. Niigata 129gou belongs to ae mutation rice cultivar, that is, 1.4 times higher value than that of the high-amylose rice, Koshinokaori; 1.1 times higher than that in the *Japonica-Indica* hybrid rice, Hoshiyutaka; 1.3~1.6 times higher than that of the high-quality premium *Japonica* rice and ordinary *Japonica* rice, Koshihikari and Kinuhikari. The Fb_3_ (DP ≧ 37) % ratios of proportion of longer amylopectin chains was higher in the *ae* mutant rice cultivars than in the other rice cultivars. The proportion of short chains was much lower in the ae mutants rice cultivars than in the other rice cultivars [33].

Amylose is one component of starch which greatly affects the quality and gelatinization properties of rice [62,63]. Apparent amylose content (AAC) comprises a large amount of amylose and a small amount of super long chain (SLC) of amylopectin. Igarashi et al. reported a positive correlation between absorbance at λmax and AAC [61]. Asaoka et al. showed that the high-temperature-ripened rice grains contained decreased levels of amylose and long chain amylopectin [64]. And Kobayashi et al. showed that amylose content decreased in Koshihikari ripened under high temperature in 2017 [65,66]. In our previous paper, the high-temperature damage for low-amylose rice was much more remarkable than ordinary *Japonica* rice, for example in amylose content, α-amylase activity and pasting property [15].

Potato starch has a higher concentration of phosphate than the starches from other botanical sources [67], and phosphorus are mainly bound to C6 of glucose residues of amylopectin [20]. It was reported that the addition of cations, such as calcium and magnesium, effectively improved physical properties of potato starch [68] by binding to the phosphate residues because potato starch has more phosphate residues than the other starches [69]. Schwall et al. created a new variety of genetically modified potato of which amylose content was very high due to low activities of starch branching enzymes [70], and it was revealed that the phosphorus content of the starch was increased more than fivefold. They pointed out that the unique potato starch, with its high amylose, low amylopectin, and high phosphorus levels, would offer novel properties for food and industrial application [70].

### 3.3. Pasting Properties of Rice Starch Using 7 Kinds of Rice Cultivars

As we measured phosphorus contents and starch molecular structures in various rice samples, we measured the pasting properties of starch using the same rice samples for investigating the relationship between phosphorus contents of the starch and calcium included in hard water. Therefore, we used Contrex (hardness: 1468 mg/L, pH: 7.2) or the purified water for the pasting property test and compared the results. To eliminate the effects of proteins, lipids, and enzymes, we used rice starch in the place of milled rice.

As shown in Table 4, Min. vis (minimum viscosity), BD (breakdown), Cons and Set/Cons of pasting properties of various kinds of starch using purified water and hard water, of which almost all samples in Contrex showed lower values than those in purified water. In contrast, Max/Fin, Pt (gelatinization temperature) and Peak time (Peak.t) of all samples in Contrex showed a little higher than those in the purified water. In many cases, high calcium contents lowered the viscosity in the pasting quality test.

The gelatinization property is one of the most important rheological indicators for the cooking quality or processing suitability of rice starch [37]. Many investigations have shown that the rheological properties of starch, such as gelatinization, retrogradation, and pasting properties, are affected by amylopectin structure [16]. The Fin. vis of high-amylose rice cultivars have been shown to be higher than those of low-amylose rice cultivars, and Fin. vis are closely related to the degree of starch retrogradation after cooling [37]. A highly positive relationship was observed between SLC content and consistency (Cons) (= Fin. vis − Mini. vis) [39].

**Figure 2:**
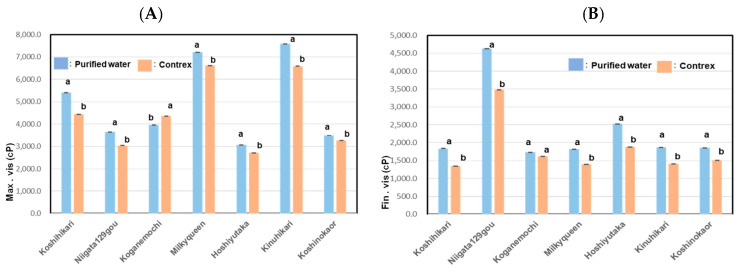
Pasting properties with maintenance temperature 120 °C of RVA for various kinds of starches using purified water or Contrex. Different letters (a, b) mean that values were significantly different. (**A**); Max. vis of 7 kinds of rice starches using purified water or Contrex with an RVA at 120 °C. (**B**); Fin. vis of 7 kinds of rice starches using purified water or Contrex with an RVA at 120 °C.

As shown in Figure 2A, Max. vis (maximum viscosity) of various kinds of starch using hard water showed lower values than those in purified water, e.g., Koshihikari showed 0.82 times, Niigata129gou; 0.83 times, Kinuhikari; 0.87 times, Hoshiyutaka; 0.88 times, Milky Queen; 0.92 times, and Koshinokaori; 0.94 times. On the other hand, that of Koganemochi showed values 1.1 times higher than that in the case of purified water.

**Figure 3:**
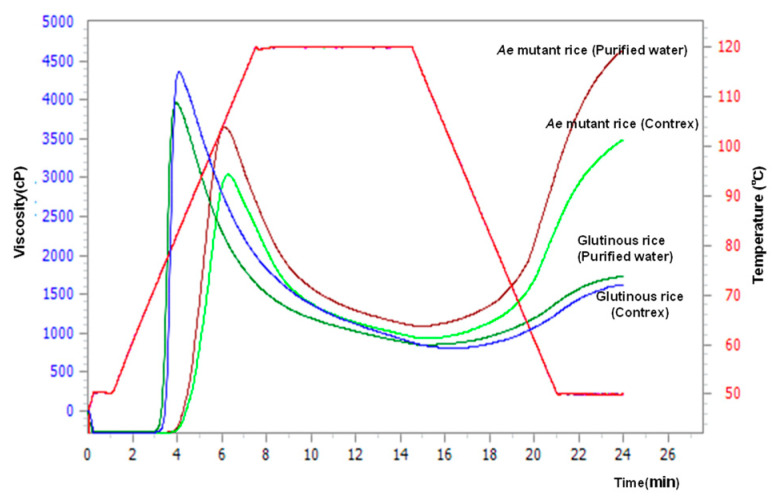
Comparison of pasting properties of 2 kinds of starch samples using purified water or Contrex with an RVA at 120 °C.

As shown in Figure 2B, the Fin. vis, indicator of retrogradation, of Niigata129gou was the highest, and that of Hoshiyutaka was the next highest among all the *Japonica* rice samples. In addition, the Fin. vis of all samples in Contrex showed 0.74~0.94 times lower than those in purified water and Mini. vis (minimum viscosity) and Cons (consistency) showed a similar tendency. Generally, high-amylose rice starches tend to retrograde more rapidly after boiling than ordinary rice and low-amylose rice [16]. Pt (gelatinization temperature) and Peak time (Peak.t) of all samples in Contrex showed a little higher than those in the purified water. RVA profiles are shown in Figure 3.

It was shown that high contents of calcium lowered Max. vis and Fin. vis except Max. vis of waxy rice. We estimate that calcium lowers viscosity because calcium binds with phosphorus in the starch, of which structure became more consistent.

It seems that the amylopectin with long-chain glucans, which consists of a helical structure in solution showed stronger binding power between phosphorus and calcium than that of amylopectin with short-chain glucans. It seems that *ae* mutant *Japonica* rice may have been influenced by cations included in Contrex, which may lead to binding to the phosphate.

Supplementary Figure S2A–C showed a similar tendency with the results mentioned above. In addition, as shown in Supplementary Figure S2D–F, pasting properties with maintenance temperature 120 °C program of RVA of polished rice using hard water and purified water showed almost the same tendency as those of pasting properties using starch.

In the present study, we showed that phosphorus contents of rice samples revealed significant correlation with sunlight hours. Moreover, high ripening temperature have a strong influence on the regulation of genes for starch synthases and branching enzymeIIb [14], which leads to decrease in the amylose content, in contrast, increase of long chain-enriched amylopectin [13].

As shown in Table 5, we evaluated the correlation between the pasting properties, phosphorus contents and iodine absorption curve of 7 various kinds of rice cultivars.

The phosphorus content showed a positive correlation with Fin. vis (r = 0.61; *p* < 0.05), Pt (r = 0.81; *p* < 0.01), Cons (r = 0.63; *p* < 0.05), AAC (r = 0.75; *p* < 0.01), Aλ_max_ (r = 0.81; *p* < 0.01), and Fb_3_ (r = 0.81; *p* < 0.01) and negative correlation with Max/Fin (r = −0.59; *p* < 0.05) and λ_max_/Aλ_max_ (r = −0.73; *p* < 0.01), which are thought to be good indexes for the retrogradation ratio of rice starches. In addition, we evaluated the correlation of the inositol contents with pasting properties and iodine absorption curve of seven various kinds of rice cultivars. It was shown that inositol had almost no correlation with pasting properties and starch microstructures. Although inositol was reported to be an important molecule which binds with phosphorus in rice grains [18], it did not show high correlation because the contents are much lower in polished rice than in unpolished rice [18].

As a result, it was shown that the phosphorus contents have significant positive correlations with starch molecular structures which cause retrogradation in a wide-range of rice cultivars.

### 3.4. Calcium and Magnesium Contents of Polished Rice, Boiled Rice, Rice Crackers, and Rice Noodles Prepared Using Contrex or Purified Water from 7 Various Kinds of Rice Samples

As shown in Table 6, the calcium contents of 7 various kinds of polished rice showed average value of 4.7 ± 0.8 (mg/100 g), particularly, glutinous *Japonica* rice, Koganemochi showed the highest value 6.0 ± 0.2 (mg/100 g), in contrast, high-amylose cultivars, Koshinokaori 4.0 ± 0.1 (mg/100 g) or Niigata129gou 4.0 ± 0.3 (mg/100 g) showed slightly lower values, and those of magnesium contents showed a similar tendency with calcium contents in 7 various kinds of rice samples.

The calcium contents of boiled rice soaked and boiled in purified water showed higher (1.4 times–3.4 times) values than those of polished rice in 7 various kinds of samples, on the contrary their magnesium contents soaked and boiled in purified water showed lower (0.6 times–0.8 times) values than those of raw polished rice. It is thought that the reason is because magnesium ions have higher solubility than calcium ion in water [71], therefore, magnesium contents of boiled rice soaked and boiled in purified water showed lower values than those of raw polished rice.

Ogawa et al. [72,73] reported that cations were ionically combined to the pectin, cell wall decomposition products, during soaking and boiling. Shibuya et al. [74] isolated various cell wall components from different parts of rice grains of *Japonica* cultivars, and reported that their compositions and sugar linkages for these cell walls were different among the rice cultivars.

**Figure 4:**
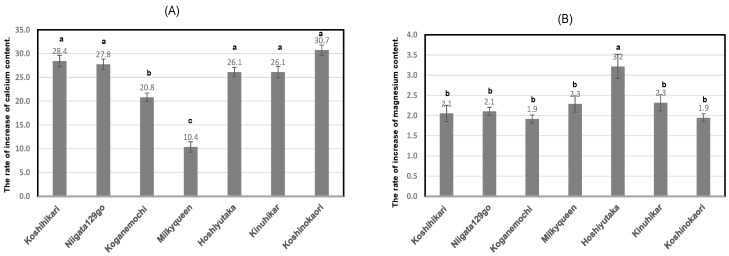
Increase rate of calcium or magnesium contents of boiled rice soaked and boiled in Contrex to those of boiled rice using purified water from 7 various kinds of rice. Different letters (a, b, c) mean that values were significantly different. (**A**); The ratio of calcium contents soaked and boiled in Contrex to those soaked and boiled in purified water using 7 various kinds of polished rice. (**B**); The ratio of magnesium contents soaked and boiled in Contrex to those soaked and boiled in purified water using 7 various kinds of polished rice.

As shown in Table 6 and Figure 4A, calcium contents of boiled rice, soaked and boiled in Contrex, showed 10.4 times–30.7 times higher than those of boiled rice soaked and boiled in purified water. Particularly, high-amylose *Japonica* rice, Koshinokaori, showed higher value than those of other cultivars. In contrast, low-amylose *Japonica* rice, Milky Queen showed the lowest value. Similarly, glutinous *Japonica* rice, Koganemochi also showed a slightly lower tendency.

As shown in Table 6 and Figure 4B, the magnesium contents in boiled rice soaked and boiled in Contrex were 1.9 times–3.2 times higher than those in boiled rice soaked and boiled in purified water. Particularly, Japonica-Indica hybrid rice, Hoshiyutaka, showed higher ratio than those of other cultivars. We found differences in Ca bindings according to the different cultivars, and this is not consistent with the report by Wariyah et al. [23]. And calcium contents of polished rice, soaked and boiled in Contrex, showed a positive correlation with those of magnesium contents (r = 0.82; *p* < 0.05).

**Figure 5:**
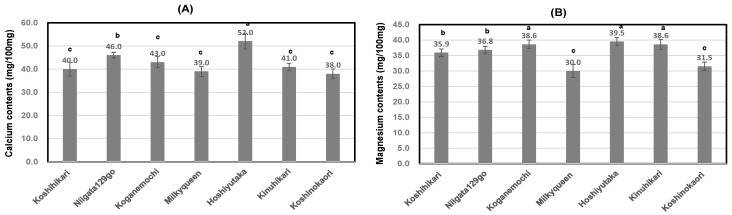
Calcium and magnesium contents of rice noodles using Contrex for 7 various kinds of polished rice flour. Different letters (a,b,c) mean that values were significantly different. (**A**); Calcium contents of rice noodles using Contrex for 7 various kinds of polished rice flour. (**B**); Magnesium contents of rice noodles using Contrex for 7 various kinds of polished rice flour.

As shown in Figure 5A, at the point of calcium contents in noodles from polished rice using Contrex, *Japonica-Indica* hybrid rice, Hoshiyutaka showed higher value than those of other cultivars. Similarly, *ae* mutant *Japonica* rice, Niigata129gou showed higher value, in contrast, low-amylose *Japonica* rice, Milky Queen showed lower value.

As shown in Figure 5B, at the point of magnesium contents in noodles from polished rice using Contrex, Hoshiyutaka showed higher value than those of other cultivars, in contrast, Milky Queen showed lower value.

As shown in Table 7, calcium in rice crackers using polished rice flour from Milky Queen, soaked and baked in Contrex (pH 4.6) or Contrex (pH 7.2), showed 8.3–8.6 times higher than those using purified water, respectively, and magnesium content of rice cracker, using Contrex (pH 7.2) or Contrex (pH 4.6), showed 1.2~1.3 times higher than those in purified water, respectively.

As above-mentioned, calcium and magnesium contents of boiled rice and rice noodles, using Contrex for soaking and boiling, showed significant correlations with λ_max_, indicator for length of amylose, and λ_max_/Aλ_max_ ratio, indicator for the ratio of amylose-to-amylopectin.

As a result, it was found that hard water like Contrex was useful for increasing the calcium absorption through the meal. Particularly, high-amylose cultivars soaked and boiled in Contrex showed higher calcium contents than those of other cultivars. In contrast, fortification effects of Ca in low-amylose rice or glutinous rice were slightly lower.

### 3.5. Textural Properties of Boiled Rice Grains Soaked and Boiled in Contrex (pH 7.2), Weakly Acid Contrex (pH 4.6) and Purified Water Using 7 Various Kinds of Polished Rice Samples

We measured and compared the textural properties of boiled rice soaked and boiled in hard water (Contrex, pH 7.2), weakly acid hard water (Contrex, pH 4.6), or purified water, using seven various kinds of rice cultivars as samples.

**Figure 6:**
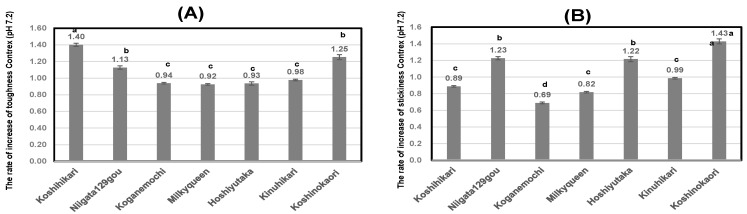
Increase rate of physical properties of boiled rice grains soaked and boiled in Contrex (pH 7.2) to those of boiled rice in purified water using 7 various kinds of polished rice cultivars. (**A**); The ratio of toughness of boiled rice soaked and boiled in Contrex (pH 7.2) to those of boiled rice soaked and boiled in purified water using 7 various kinds of polished rice. (**B**); The ratio of stickiness of boiled rice soaked and boiled in Contrex (pH 7.2) to those of boiled rice soaked and boiled in purified water using 7 various kinds of polished rice. Different letters (a, b, c, d) mean that values were significantly different.

As shown in Table 8 and Figure 6A, among seven various kinds of polished rice cultivars, the toughness of boiled rice grains soaked and boiled in Contrex (pH 7.2) was higher (Koshihikari; 1.4 times, Koshinokaori; 1.3 times, and Niigata129gou; 1.1 times) than those of boiled rice soaked and boiled in purified water. On the contrary, those of the other cultivars showed slightly lower values than those of boiled rice in the purified water.

As shown in Table 8 and Figure 6B, the stickiness of rice soaked and boiled in Contrex (pH 7.2) was higher (Koshinokaori; 1.4 times, Hoshiyutaka; 1.2 times, and Niiata129gou; 1.2 times) than those of boiled rice grains soaked and boiled in purified water, although those of the other cultivars soaked and boiled in Contrex (pH 7.2) were lower than those of boiled rice grains in purified water. Particularly, glutinous *Japonica* rice, Koganemochi (0.7 times), low-amylose *Japonica* rice, Milky Queen (0.8 times) showed lower values.

As a result, we found that, among the various kinds of polished rice soaked and boiled in Contrex (pH 7.2), toughness and stickiness of *ae* mutant rice, high-amylose rice and *Japonica-Indica* hybrid rice showed higher values than those of glutinous rice and low-amylose rice. It is thought that this is because calcium in hard water contributes to strengthening the consistent structure of the boiled rice grains, as they have high amount of phosphorus residues in the amylopectin long chains where calcium binds and strengthens the physical structure.

We reported that in addition to the activities of endogenous amylase and proteinase, cell-wall-degrading enzymes were also activated by ripening under high temperature [16].

Boiled rice became softened by the decomposition of the cell-wall, suggesting the important role of cell-wall degrading enzyme in the physical properties of the endosperm cell wall. As shown in Supplementary Figure S3, the endo-xylanase activities of seven kinds of unpolished rice grains were 0.356 ± 0.153 (Ug^−1^)(Kinuhikari; 0.605 ± 0.001 (Ug^−1^), Niigata129gou; 0.491 ± 0.001 (Ug^−1^), Milky Queen; 0.371 ± 0.002 (Ug^−1^), Koshihikari; 0.358 ± 0.001 (Ug^−1^), Koshinokaori; 0.252 ± 0.001 (Ug^−1^), Hoshiyutaka; 0.252 ± 0.001 (Ug^−1^), Koganemochi; 0.160 ± 0.001 (Ug^−1^). Particularly, Kinuhikari showed the highest value. It seems that Kinuhikari may have been damaged more than the other cultivars by high temperature.

In addition, we tried to improve the textural qualities of boiled rice using weakly acid Contrex (pH 4.6). In our previous paper, we reported that the hard (pH 7.2) water is useful for the prevention of the texture deterioration of the boiled rice grains due to the inhibition of the reduction of endogenous hydrolytic enzymes, such as amylase, proteinase, and xylanase in ordinary *Japonica* rice cultivars [16].

In this paper, we investigated the α-amylase activities of unpolished rice soaked in two kinds of hard water (pH 7.2), weakly acid hard water (pH 4.6) and purified water and those of oligosaccharide contents. As a result, weakly acid Contrex (pH 4.6) showed higher α-amylase activity levels than those of Contrex (pH 7.2), as shown in Supplementary Table S1.

As shown in Table 8, toughness of boiled rice soaked and boiled in Contrex (pH 4.6) were higher (Koshinokaori; 1.1 times, and Niigata129gou; 1.5 times) than those of boiled rice soaked and boiled in purified water, but those of the other cultivars showed lower values than those of boiled rice in purified water. Similarly, a stickiness of boiled rice soaked and boiled in Contrex (pH 4.6) showed higher (Niigata129gou; 1.2 times, Koshinokaori; 1.2 times), than those of boiled rice in purified water, but those of other cultivars showed lower values than those of boiled rice in purified water. It was shown that the texture of the boiled rice soaked and cooked in hard water was remarkably improved compared with that in purified water. And it was not reported by Wariyah et al. [23].

To summarize, toughness and stickiness of boiled rice from *ae* mutant rice and high-amylose boiled rice soaked and boiled in Contrex (pH 4.6) showed almost similar tendency to those in Contrex (pH 7.2).

It is thought that the effect, of calcium in hard water to strengthen the physical structure of the boiled rice by binding with phosphorus in starch, is preserved also in Contrex (pH 4.6), which is almost similar tendency to those in Contrex (pH 7.2).

### 3.6. Pasting Properties with Maintenance Temperature 93 °C Program of RVA for Various Kinds of Polished Rice Using Hard Water, Weakly Acid Hard Water and Purified Water

We measured the pasting properties of polished rice using instead of starch, which were influenced proteins, lipids, and hydrolytic enzymes.

As shown in Supplementary Table S2, Max. vis or Fin. vis of Koshihikari, Koganemochi, Milky Queen and Kinuhikari in Contrex (pH 7.2) were higher than those in purified water, in contrast, those of Niigata129gou, Hoshiyutaka and Koshinokaori showed lower values than those in purified water, and their Min. vis values, BD values, and Cons values showed almost similar tendency among 7 various kinds of polished rice flours.

**Figure 7:**
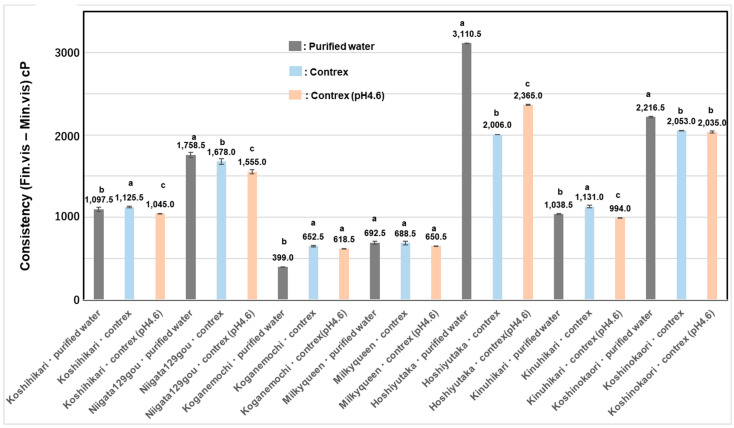
Consistency values in Contrex (pH 7.2), weakly acid Contrex (pH 4.6), or purified water using 7 various kinds of polished rice flours. Different letters (a, b, c) mean that values were significantly different.

As shown in Figure 7, cons values in Contrex (pH 7.2) or Contrex (pH 4.6) of almost all samples except Koganemochi measured lower than those in purified water, especially, Niigata129gou, Koshinokaori, and Hoshiyutaka showed markedly lower values, which showed hard water reduces retrogradation in the case of high-amylose or long-chain-rich-amylopectin rice.

As a result, pasting properties with maintenance temperature 93 °C program of RVA of polished rice using hard water measured slightly lower than those in purified water, which showed almost the same tendency as those of pasting properties using starch, as shown in Table 3.

### 3.7. Correlation among the Physico-Chemical and Biological Properties

As shown in Table 9, we evaluated the correlation among the textural properties, pasting properties, iodine absorption curve, xylanase activities and phosphorus contents of 7 various kinds of polished rice samples.

The phosphorus content showed a positive correlation with Min. vis (r = 0.56; *p* < 0.05), Set/Cons (r = 0.98; *p* < 0.01), Pt (r = 0.55; *p* < 0.05), AAC (r = 0.75; *p* < 0.01), Aλ_max_ (r = 0.81; *p* < 0.01), Fb_3_ (r = 0.81; *p* < 0.01), hardness (r = 0.67; *p* < 0.01) and negative correlation with BD (r = −0.65; *p* < 0.05), Max/Min (r = −0.69; *p* < 0.01) and Max/Fin (r = −0.68; *p* < 0.01). In addition, we evaluated the correlation among the inositol contents, textural properties, pasting properties, iodine absorption curve, xylanase activities and phosphorus contents of 7 various kinds of rice cultivars, as a result, it was shown that there were almost no correlations.

Furthermore, Fb_3_ (DP ≧ 37) % ratios of proportion of longer amylopectin chains showed a positive correlation with hardness (r = 0.77; *p* < 0.01), toughness (r = 0.54; *p* < 0.05), stickiness (r = 0.59; *p* < 0.05), phosphorus content (r = 0.81; *p* < 0.01), M in. vis (r = 0.67; *p* < 0.01), Fin (r = 0.80; *p* < 0.01), Pt (r = 0.67; *p* < 0.01), Cons (r = 0.69; *p* < 0.01) and Set/Cons (r = 0.98; *p* < 0.01) and negative correlation with BD (r = −0.69; *p* < 0.01), Max/Min (r = −0.77; *p* < 0.01) and Max/Fin (r = −0.87; *p* < 0.01).

Takeda and Hizukuri reported that potato starch has a larger amount of covalently bound phosphate in its amylopectin component, and the phosphate groups are located at C6 and C3 of the glucosyl residues [75]. Moreover, wheat starch granules contain about 1% lipid, and the phosphorus is in the form of lysolecithin, some of which are complexed with amylose or outer chains of amylopectin as helical complexes [76,77,78]. Some of the phosphate esters on adjacent amylopectin chains are naturally found as cross-linked with various cations, such as calcium and magnesium [79,80]. The substitution of cations from hydrogen ions, etc., to calcium bound to the phosphate was carried out for the purpose of changing the physical properties of potato starch [69,80,81].

The toughness of boiled rice showed a positive correlation with Pt (r = 0.65; *p* < 0.05) and Set/Cons (r = 0.95; *p* < 0.01), and hardness (r = 0.65; *p* < 0.05). Moreover, the stickiness showed positive correlation with Fin. vis (r = 0.59; *p* < 0.01), Cons (r = 0.64; *p* < 0.05), Set/Cons (r = 0.86; *p* < 0.01) and toughness (r = 0.66; *p* < 0.05).

A novel method was developed to maintain the good texture by inhibiting excess activities of glycolytic enzymes and cell-wall degrading enzymes during the boiling and binding phosphorus contained in starch and cations contained in hard water.

As a result, the proportion of long chains and intermediate- and long chains of amylopectin showed a positive correlation with toughness and stickiness of boiled rice.

The endo-xylanase activities showed a positive correlation with Pt (r = 0.72; *p* < 0.01) and Set/Cons (r = 0.98; *p* < 0.01).

As a result, endo-xylanase activities and phosphorus content showed a positive correlation with the proportion of long chains of amylopectin.

AAC of iodine absorption spectrum showed a positive correlation with hardness (r = 0.76; *p* < 0.01), toughness (r = 0.57; *p* < 0.05), stickiness (r = 0.65; *p* < 0.05) and Aλ_max_ showed a similar tendency.

As a result, proportion of longer amylopectin chains showed a high positive correlation with phosphorus content, therefore toughness and stickiness of boiled rice showed a high positive correlation with AAC and Aλ_max_ of starch characteristics by cations contained in Contrex bound to the phosphate of glucose residue of amylopectin.

In conclusion of the statistical treatment, it seems a novel finding that phosphorus contents showed significant correlations with textural property such as toughness and stickiness pasting properties such as Pt and Set/Cons, and starch microstructure such as AAC and Fb_3_.

It would be thought that cations, such as calcium, make ionic binding with phosphorus residues rich in amylopectin long chains, which would strengthen the textural and pasting properties. This hypothesis is harmonized well with the binding of cations with phosphorus contained in starch as reported by Lin et al. [78].

### 3.8. Improvement of the Color of Boiled Rice by Using Weakly Acidic Hard Water, Contrex (pH 4.6)

Generally, it is said that boiled rice grains cooked in hard water show harder texture, with more yellowish color than rice cooked in soft water [72]. It is well-known that color differences of boiled rice are affected by amino-carbonyl reactions of reducing sugar with amino acids. It is well-known that color differences in boiled rice are affected by amino-carbonyl reactions of reducing sugar with amino acids.

As shown in Table 10, we evaluated color difference of gelatinizing paste or boiled rice soaked and boiled in 2 kinds of hard water (Evian or Contrex) or those of weakly acidic hard water (pH 4.6) compared with the boiled rice grains soaked and boiled in purified water. Yellowish degree (ratio of color difference b*) of boiled rice soaked and boiled in weakly acidic hard water (Contrex pH 4.6 or Evian pH 4.6) showed (0.4 times or 0.6 times) lower b* values compared to the rice grains soaked and boiled in purified water, respectively, and those of gelatinized paste showed a similar tendency. Moreover, a ratio of the color difference (ΔE*(ab)) of boiled rice grains or gelatinized paste showed a similar tendency as b* values.

Also, reddish degree (ratio of color difference a*) of boiled rice soaked and boiled in weakly acidic hard water (pH 4.6) showed slightly lower compared to the rice grains soaked and boiled in purified water.

### 3.9. Sensory Evaluation of Boiled Rice or Rice Noodles Soaked and Boiled in 2 Kinds of Hard Water, or Weakly Acidic Hard Water, or Purified Water

**Figure 8:**
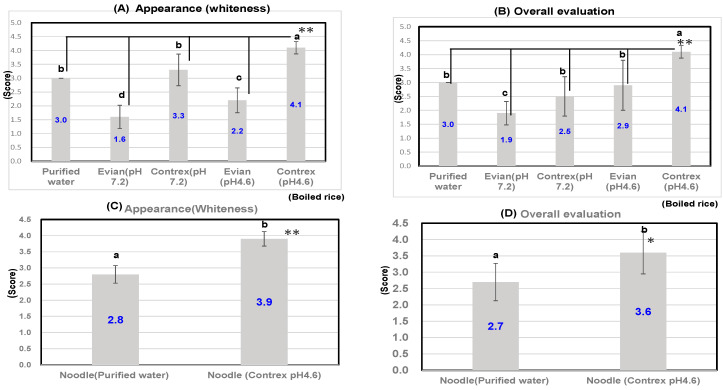
Sensory test of boiled rice or rice noodles soaked and boiled in the various kinds of water. Means with same letter are not significantly different. By 10 panelists. Correlation is significant at 5% (*) or 1% (**) by the method of *t*-test. ** *p* < 0.01, * *p* < 0.05. (**A**,**B**); Boiled rice samples were prepared using Evian (pH 7.2), Contrex (pH 7.2), Evian (pH 4.6), Contrex (pH 4.6), and purified water from Tennotsubu. (**C**,**D**); Rice noodle samples were prepared using Contrex (pH 4.6) or the purified water from Milky Queen.

As shown in Figure 8A, we carried out sensory evaluation of boiled rice using polished rice of Tennotsubu soaked and boiled in two kinds of hard water (Evian, Contrex pH 7.2) or those of weakly acidic hard water (pH 4.6) or purified water. As a result, appearance of boiled rice soaked and boiled in Contrex (pH 4.6) showed significantly higher scores than those of soaked and boiled in purified water, Evian (pH 7.2), Contrex (pH 7.2), and Evian (pH 4.6) at the level of 1%, and those of overall evaluation of boiled rice soaked and boiled in Contrex (pH 4.6) showed similar better evaluations, as shown in Figure 8B.

As shown in Figure 8C, we carried out sensory evaluation of rice noodles using polished rice flour of Milky Queen soaked and boiled in Contrex (pH 4.6) or purified water. As a result, appearance of rice noodles soaked and boiled in Contrex (pH 4.6) showed significantly better scores than that of soaked and boiled in purified water at the level of 1%, and those of overall evaluation of rice noodles soaked and boiled in Contrex (pH 4.6) showed similar better scores as shown in Figure 8D.

To summarize, it became possible to prepare the boiled rice or rice noodle of high qualities in terms of texture and appearance qualities by soaking and boiling in Contrex (pH 4.6) even though using the high-temperature-damaged grains.

## 4. Conclusions

We used various kinds of rice cultivars, from waxy to high-amylose ones, for soaking and boiling in hard water and compared physical and chemical properties of the products.

It was shown that the degree of quality improvement, determined by parameters such as final viscosities in pasting property and textural properties of boiled rice, was more remarkable for high-amylose rice than low-amylose rice. As we found that the phosphorus contents showed positive correlations with amylose and long chains of amylopectin, we estimate that the effects are mainly due to binding of calcium, in hard water, and phosphorus in starch.

These findings provide a scientific basis for the quality improvement of high-temperature-damaged rice grains by using hard water for cooking, consequently leading to the development of rice products fortified with calcium.

## Figures and Tables

**Table 1:** Material rice samples.

Unpolished Rice			Polished Rice
Ordinary-*Japonica* Rice	High-Quality Premium *Japonica* Rice	Low-Amylose *Japonica* Rice	Various Kinds of Rice
Haenuki (Yamagata)	Koshihikari (Saga)	Yumepirika (Hokkaidou a)	Koshihikari (Niigata)
Sasanishiki (Miyagi)	Koshihikari (Ibaraki a)	Yumepirika (Hokkaidou b)	Niigata129gou (Niigata)
Yuudai21 (Tochigi)	Koshihikari (Ibaraki b)	MilkyQueen (Yamagata)	Koganemochi (Niigata)
Ginnoshizuku (Iwate)	Koshihikari (Shimane)	Milkyqueen (Kyoto)	Milkyqueen (Niigata)
Kazesayaka (Nagano)	Koshihikari (Niigata a)		Hoshiyutaka (Niigata)
Tugaruroman (Aomori)	Koshihikari (Niigata b)		Kinuhikari (Hyogo)
Hatsushimo (Gifu)	Koshiibuki (Niigata c)		Koshinokaori (Niigata)
Aichinokaori (Aichi)	Koshihikari (Yamagata a)		Tennotsubu (Fukushims)
Gohyakukawa (Yamanashi)	Koshihikari (Yamagata b)		
Akitakomachi (Ibaraki)	Koshihikari (Ishikawa)		
Akitakomachi (Chiba)	Koshihkari (Yamanashi)		
Akitakomachi (Akita a)			
Akitakomachi (Akita b)			
Tsuyahime (Shimane)			
Tsuyahime (Yamagata a)			
Tsuyahime (Yamagata b)			
Tsuyahime (Miyagi)			

**Table 2:** Phosphorus contents of 7 various kinds of polished rice samples.

	Phosphorus Contents (mg/100 g)
Koshihikari	89.1 ± 2.6 ^b^
Niigata129gou	112.8 ± 1.5 ^a^
Koganemochi	91.8 ± 3.2 ^b^
Milkyqueen	89.1 ± 3.0 ^b^
Hoshiyutaka	88.5 ± 2.1 ^b^
Kinuhikari	90.3 ± 1.8 ^b^
Koshinokaori	110.0 ± 2.7 ^a^

Different letters (^a, b^) denote statistically significant difference. Values are shown as mean ± standard deviation.

**Table 3:** The iodine absorption curve of starch of 7 various kinds of rice.

Cultivars	AAC (%)	λ_max_	A_λmax_	λ_max_/ A_λmax_	Fb_3_ (DP ≥ 37) (%)
Koshihikari	15.9 ± 0.1 ^c^	580.5 ± 0.7 ^c^	0.310 ± 0.00 ^c^	1872.6 ± 2.6 ^c^	13.1 ± 0.0 ^d^
Niigata129gou	39.2 ± 0.6 ^a^	592.0 ± 1.0 ^a^	0.620 ± 0.01 ^a^	958.2 ± 18.8 ^f^	26.1 ± 0.3 ^a^
Koganemochi	4.0 ± 0.1 ^f^	523.0 ± 0.0 ^f^	0.250 ± 0.00 ^e^	2087.0 ± 10.7 ^b^	10.2 ± 0.1 ^e^
Milkyqueen	10.1 ± 0.4 ^e^	533.5 ± 3.5 ^e^	0.196 ± 0.00 ^f^	2729.2 ± 31.3 ^a^	8.0 ± 0.2 ^f^
Hoshiyutaka	25.2 ± 0.6 ^b^	595.8 ± 1.8 ^b^	0.405 ± 0.01 ^b^	1430.5 ± 24.5 ^d^	17.6 ± 0.3 ^c^
Kinuhikari	13.9 ± 0.4 ^d^	573.0 ± 1.4 ^d^	0.271 ± 0.00 ^d^	2118.3 ± 0.3 ^b^	11.3 ± 0.0 ^e^
Koshinokaori	25.8 ± 0.1 ^b^	596.0 ± 0.0 ^b^	0.446 ± 0.00 ^b^	1337.8 ± 2.1 ^e^	19.1 ± 0.0 ^b^

Different letters (^a–f^) denote statistically significant difference in the same column (AAC, λ_max_, etc.). Values are shown as mean ± standard deviation.

**Table 4:** Pasting properties with maintenance temperature 120 °C program of RVA for various kinds of starch using purified water or hard water.

Cultivars/Water Types	Mini.vis (cP)	BD (cP)	SB (cP)	Pt (°C)	Cons (cP)	Set/Cons	Max/Min	Max/Fin	Peak.t (min)
Koshihikari (Purified water)	484.0 ± 3.7 ^a^	4926.0 ± 9.7 ^a^	−3574.0 ± 1.1 ^b^	75.1 ± 0.0 ^b^	1352.0 ± 5.5 ^a^	−2.6 ± 0.0 ^a^	11.2 ± 0.1 ^a^	2.9 ± 0.0 ^b^	5.5 ± 0.0 ^b^
Koshihikari (Contrex)	392.0 ± 2.1 ^b^	4052.0 ± 0.6 ^b^	−3091 ± 2.2 ^a^	77.6 ± 0.1 ^a^	961.0 ± 1.9 ^b^	−3.2 ± 0.0 ^b^	11.3 ± 0.0 ^a^	3.3 ± 0.0 ^a^	6.0 ± 0.1 ^a^
Niigata129gou (Purified water)	1088.0 ± 0.4 ^a^	2561.0 ± 5.1 ^a^	−978.0 ± 1.1 ^b^	85.1 ± 0.0 ^b^	3539.0 ± 1.8 ^a^	−0.3 ± 0.0 ^a^	3.4 ± 0.0 ^a^	0.8 ± 0.0 ^a^	6.1 ± 0.0 ^a^
Niigata129gou (Contrex)	934.0 ± 0.1 ^b^	2104.0 ± 0.6 ^b^	−440.0 ± 1.7 ^a^	86.7 ± 0.0 ^a^	2544.0 ± 0.3 ^b^	−0.2 ± 0.0 ^a^	3.3 ± 0.2 ^a^	0.9 ± 0.0 ^a^	6.3 ± 0.0 ^a^
Koganemochi (Purified water)	849.0 ± 1.9 ^a^	3117.0 ± 0.6 ^b^	−2238.0 ± 3.0 ^a^	75.2 ± 0.0 ^b^	879.0 ± 0.1 ^a^	−2.5 ± 0.0 ^a^	4.7 ± 0.0 ^b^	2.3 ± 0.0 ^b^	3.9 ± 0.0 ^a^
Koganemochi (Contrex)	807.0 ± 1.4 ^b^	3553.0 ± 1.8 ^a^	−2738.0 ± 0.5 ^b^	76.6 ± 0.0 ^a^	815.0 ± 0.6 ^b^	−3.4 ± 0.0 ^b^	5.4 ± 0.0 ^a^	2.7 ± 0.0 ^a^	4.1 ± 0.0 ^a^
Milkyqueen (purified water)	690.0 ± 3.7 ^a^	6528.0 ± 9.7 ^a^	−5401.0 ± 2.2 ^a^	74.4 ± 0.0 ^a^	1127.0 ± 5.5 ^a^	−4.8 ± 0.0 ^a^	10.5 ± 0.1 ^b^	4.0 ± 0.0 ^b^	5.2 ± 0.0 ^a^
Milkyqueen (Contrex)	523.0 ± 2.1 ^b^	6101.0 ± 0.6 ^b^	−5220.0 ± 1.1 ^a^	74.8 ± 0.1 ^a^	881.0 ± 1.9 ^b^	−5.9 ± 0.0 ^b^	12.7 ± 0.0 ^a^	4.7 ± 0.0 ^a^	5.5 ± 0.1 ^a^
Hoshiyutaka (Purified water)	669.0 ± 2.1 ^a^	2395.0 ± 0.6 ^a^	−533.0 ± 2.2 ^a^	66.2 ± 0.1 ^a^	1862.0 ± 1.9 ^a^	−0.3 ± 0.0 ^a^	4.6 ± 0.0 ^a^	1.2 ± 0.0 ^a^	5.9 ± 0.1 ^a^
Hoshiyutaka (Contrex)	639.0 ± 0.4 ^b^	2071.0 ± 5.1 ^b^	−826.0 ± 1.1 ^b^	67.4 ± 0.0 ^a^	1245.0 ± 1.8 ^b^	−0.7 ± 0.0 ^b^	4.2 ± 0.0 ^a^	1.4 ± 0.0 ^a^	6.1 ± 0.0 ^a^
Kinuhikari (Purified water)	1086.0 ± 2.1 ^a^	6504.0 ± 0.6 ^a^	−5715.0 ± 1.1 ^b^	70.2 ± 0.1 ^b^	789.0 ± 1.9 ^b^	−7.2 ± 0.0 ^b^	7.0 ± 0.0 ^b^	4.0 ± 0.0 ^a^	5.4 ± 0.1 ^b^
Kinuhikari (Contrex)	868.8 ± 0.4 ^b^	5203.2 ± 5.1 ^b^	−4857.8 ± 1.7 ^a^	71.8 ± 0.0 ^a^	560.2 ± 1.8 ^a^	−8.8 ± 0.0 ^a^	7.0 ± 0.0 ^a^	4.6 ± 0.0 ^b^	5.9 ± 0.0 ^a^
Koshinokaori (Purified water)	539.0 ± 1.9 ^a^	2945.0 ± 0.6 ^a^	−1626.0 ± 3.0 ^a^	78.6 ± 0.0 ^a^	1319.0 ± 5.5 ^a^	−1.2 ± 0.0 ^a^	6.5 ± 0.1 ^a^	1.9 ± 0.0 ^a^	5.5 ± 0.0 ^a^
Koshinokaori (Contrex water)	503.0 ± 1.4 ^a^	2755.0 ± 1.8 ^b^	−1747.0 ± 0.5 ^b^	80.0 ± 0.1 ^a^	1008.0 ± 1.9 ^b^	−1.7 ± 0.0 ^b^	6.5 ± 0.0 ^a^	2.2 ± 0.0 ^a^	5.8 ± 0.1 ^a^

Different letters (^a, b^) mean that values were significantly different.

**Table 5:** Correlation between the pasting properties, phosphorus contents and iodine absorption curve of 7 various kinds of rice starch samples.

	Max	Mini	BD	Fin	Pt	Cons	Max/ Min	Max/ Fin	Peak.T	Phosph orus	AAC	λ_max_	A_λmax_	λ_max_/ A_λmax_	Fb_3_
Max.vis	1.00														
Mini.vis	0.14	1.00													
BD	0.99 **	0.00	1.00												
Fin.vis	−0.39	0.59 *	−0.45	1.00											
Pt	−0.27	0.16	−0.28	0.52	1.00										
Cons	−0.47	0.39	−0.51	0.97 **	0.55 *	1.00									
Max/Mini	0.64 *	−0.61 *	0.75 **	−0.58 *	−0.15	−0.49	1.00								
Max/Fin	0.90 **	−0.16	0.92 **	−0.68 **	−0.33	−0.73 **	0.76 **	1.00							
Peak. T	−0.19	−0.10	−0.20	0.38	0.13	0.46	−0.03	−0.24	1.00						
Phosphorus	−0.50	0.23	−0.53	0.61 *	0.81 **	0.63 *	−0.48	−0.59 *	0.31	1.00					
AAC	−0.59	0.19	−0.62 *	0.77 **	0.47	0.82 **	−0.51	−0.73 **	0.77 **	0.75 **	1.00				
λ_max_	−0.49	−0.10	−0.49	0.36	0.08	0.44	−0.31	−0.53	0.82 **	0.45	0.81 **	1.00			
Aλ_max_	−072 **	0.26	−0.76 **	0.80 **	0.56 *	0.84 **	−0.66 *	−0.85 **	0.59 *	0.81 **	0.96 **	0.73 **	1.00		
λ_max_/Aλ_max_	0.81 **	−0.17	0.85 **	−0.66 **	−0.43	−0.71 **	0.71 **	0.89 **	−0.51	−0.73 **	−0.88 **	−0.79 **	−0.96 **	1.00	
Fb_3_	−0.73 **	0.24	−0.77 **	0.78 **	0.54 *	0.83 **	−0.66 *	−0.85 **	0.60 *	0.81 **	0.96 **	0.75 **	1.00 **	−0.96 **	1.00

Correlation is significant at 5% (*) or 1% (**) by the method of *t*-test.

**Table 6:** Calcium and magnesium contents of polished rice and boiled rice soaked and boiled in purified water or Contrex using 7 various kinds of rice.

	Cultivar	Calcium	Magnesium
		(mg/100 g)	(mg/100 g)
Polished rice	Koshihikari	5.0 ± 0.2 ^b^	25.0 ± 0.3 ^b^
	Niigata129go	4.0 ± 0.3 ^c^	26.0 ± 0.2 ^b^
	Koganemochi	6.0 ± 0.2 ^a^	31.0 ± 0.1 ^a^
	Milkyqueen	5.0 ± 0.3 ^b^	22.0 ± 0.2 ^c^
	Hoshiyutaka	5.0 ± 0.2 ^b^	19.0 ± 0.1 ^c^
	Kinuhikar	4.0 ± 0.2 ^c^	24.0 ± 0.2 ^b^
	Koshinokaori	4.0 ± 0.1 ^c^	22.0 ± 0.3 ^b^
Purified water (boiled rice)	Koshihikari	7.0 ± 0.4 ^c^	20.0 ± 0.4 ^b^
	Niigata129go	8.0 ± 0.5 ^c^	20.0 ± 0.4 ^b^
	Koganemochi	10.0 ± 0.6 ^b^	23.0 ± 0.4 ^a^
	Milkyqueen	17.0 ± 1.1 ^a^	14.0 ± 0.3 ^c^
	Hoshiyutaka	10.0 ± 0.6 ^b^	14.0 ± 0.3 ^c^
	Kinuhikar	7.0 ± 0.4 ^c^	19.0 ± 0.4 ^b^
	Koshinokaori	8.0 ± 0.5 ^c^	18.0 ± 0.3 ^b^
Contrex (boiled rice)	Koshihikari	199.0 ± 12.9 ^c^	41.0 ± 1.6 ^b^
	Niigata129go	222.0 ± 14.4 ^b^	42.0 ± 1.5 ^b^
	Koganemochi	208.0 ± 13.5 ^c^	44.0 ± 1.6 ^a^
	Milkyqueen	176.0 ± 11.4 ^d^	32.0 ± 1.4 ^c^
	Hoshiyutaka	261.0 ± 17.0 ^a^	45.0 ± 1.3 ^a^
	Kinuhikari	215.0 ± 14.0 ^b^	44.0 ± 1.6 ^a^
	Koshinokaori	178.0 ± 11.6 ^d^	35.0 ± 1.5 ^c^

Different letters (^a–d^) mean that values were significantly different. Values are shown as mean ± standard deviation.

**Table 7:** Calcium and magnesium contents in rice crackers and rice noodles using polished rice flour of Milky Queen soaked in Contrex (pH 7.2), weakly acid Contrex (pH 4.6) and purified water.

	Calcium	Magnesium
	(mg/100 g)	(mg/100 g)
Rice crackers (Purified water)	6.1 ± 0.0 ^b^	27.5 ± 0.0 ^b^
Rice crackers (Contrex) (pH 7.2)	52.2 ± 0.0 ^a^	34.3 ± 0.0 ^a^
Rice crackers (Contrex) (pH 4.6)	50.5 ± 0.0 ^a^	35.3 ± 0.0 ^a^
Noodle (Purified water)	6.3 ± 0.4 ^b^	26.7 ± 0.5 ^b^
Noodle (Contrex) (pH 7.2)	37.5 ± 3.5 ^a^	30.0 ± 2.8 ^a^
Noodle (Contrex) (pH 4.6)	35.4 ± 2.3 ^a^	31.4 ± 1.9 ^a^

Different letters (^a, b^) mean that values were significantly different. Values are shown as mean ± standard deviation.

**Table 8:** Textural properties of boiled rice after soaking in Contrex, weakly acid Contrex and purified water using 7 various kinds of polished rice.

	Hardness	Toughness	Adhesion	Stickiness
	×10^5^ (N/cm^2^)	×10^5^ (N/cm^2^)	×10^5^ (N/cm^2^)	×10^5^ (N/cm^2^)
Koshihikari ⋅ purified water	0.00 ±0.00 ^a^	1.90 ± 0.07 ^b^	40.60 ± 0.06 ^b^	28.71 ± 0.79 ^a^
Koshihikari ⋅ contrex	0.01 ± 0.01 ^a^	2.66 ± 0.05 ^a^	28.17 ± 1.28 ^c^	25.46 ± 1.78 ^b^
Koshihikari ⋅ contrex (pH 4.6)	0.00 ± 0.00 ^a^	1.03 ± 0.04 ^b^	50.34 ± 1.42 ^a^	11.69 ± 1.28 ^c^
Niigata129gou ⋅ purified water	0.71 ± 0.00 ^b^	7.78 ± 2.08 ^b^	55.61 ± 0.51 ^b^	41.00 ± 0.19 ^b^
Niigata129gou ⋅ contrex	0.82 ± 0.00 ^a^	8.77 ± 0.94 ^b^	57.80 ± 0.34 ^b^	50.26 ± 0.62 ^a^
Niigata129gou ⋅ contrex (pH 4.6)	0.48 ± 0.00 ^c^	11.80 ± 0.94 ^a^	69.57 ± 0.25 ^a^	51.06 ± 0.22 ^a^
Koganemochi ⋅ purified water	0.00 ± 0.02 ^a^	1.29 ± 0.22 ^a^	40.83 ± 0.15 ^c^	19.14 ± 1.08 ^a^
Koganemochi ⋅ contrex	0.00 ± 0.01 ^a^	1.21 ± 0.12 ^a^	79.71 ± 0.00 ^b^	13.19 ± 2.21 ^b^
Koganemochi ⋅ contrex(pH 4.6)	0.02 ± 0.01 ^a^	1.09 ± 0.02 ^b^	128.52 ± 0.32 ^a^	10.34 ± 2.21 ^b^
Milkyqueen ⋅ purified water	0.00 ± 0.01 ^a^	2.37 ± 0.33 ^a^	30.99 ± 1.28 ^a^	26.18 ± 3.81 ^a^
Milkyqueen ⋅ contrex	0.00 ± 0.00 ^a^	2.19 ± 0.25 ^a^	25.23 ± 1.03 ^c^	21.40 ± 2.01 ^b^
Milkyqueen ⋅ contrex (pH 4.6)	0.00 ± 0.00 ^a^	1.46 ± 0.15 ^b^	27.02 ± 1.03 ^b^	16.71 ± 1.01 ^c^
Hoshiyutaka ⋅ purified water	0.03 ± 0.00 ^a^	4.44 ± 0.37 ^a^	47.28 ± 1.27 ^b^	54.08 ± 1.54 ^b^
Hoshiyutaka ⋅ contrex	0.02 ± 0.00 ^a^	4.15 ± 0.22 ^a^	51.20 ± 1.42 ^a^	65.73 ± 3.40 ^a^
Hoshiyutaka ⋅ contrex (pH 4.6)	0.00 ± 0.00 ^a^	1.92 ± 0.22 ^b^	47.39 ± 0.32 ^b^	35.31 ± 1.40 ^c^
Kinuhikari ⋅ purified water	0.00 ± 0.01 ^a^	1.81 ± 0.06 ^a^	39.08 ± 0.47 ^b^	21.34 ± 0.35 ^a^
Kinuhikari ⋅ contrex	0.00 ± 0.00 ^a^	1.77 ± 0.08 ^a^	43.35 ± 0.57 ^a^	21.03 ± 0.36 ^a^
Kinuhikari ⋅ contrex (pH 4.6)	0.00 ± 0.00 ^a^	1.68 ± 0.18 ^a^	44.97 ± 0.57 ^a^	19.29 ± 0.26 ^a^
Koshinokaori ⋅ purified water	0.06 ± 0.00 ^b^	7.88 ± 2.12 ^b^	43.56 ± 14.27 ^a^	34.06 ± 1.88 ^b^
Koshinokaori ⋅ contrex	0.09 ± 0.00 ^a^	9.87 ± 2.05 ^a^	41.38 ± 16.98 ^a^	48.64 ± 1.90 ^a^
Koshinokaori ⋅ contrex (pH 4.6)	0.05 ± 0.00 ^b^	8.38 ± 1.03 ^a^	43.82 ± 16.98 ^a^	43.77 ± 1.90 ^a^

Different letters (^a–c^) mean that boiled rice in each sample was significantly different. Values are shown as mean ± standard deviation.

**Table 9:** Correlation between the textural properties, phosphorus contents, xylanase activities and iodine absorption curve of 7 various kinds of polished rice samples.

	**Max**	**Mini**	**BD**	**Fin**	**Pt**	**Cons**	**Set/** **Cons**	**Max/** **Min**	**Max/** **Fin**		
Hardness	−0.48	0.66 **	−0.64 *	0.40	0.84 **	0.19	0.96 **	−0.65 *	−0.53 *		
Toughness	−0.04	0.44	−0.16	0.34	0.65 *	0.22	0.95 **	−0.21	−0.25		
Adhesion	−0.57 *	0.15	−0.57 *	0.10	0.03	0.05	0.90 **	−0.57 *	−0.51		
Stickiness	−0.14	0.24	−0.20	0.59 *	0.43	0.64 *	0.86 **	−0.22	−0.46		
xylanase	0.50	0.51	0.31	0.12	0.72 **	−0.10	0.98 **	0.13	0.23		
Phosphorus	−0.52	0.56 *	−0.65 *	0.51	0.55 *	0.38	0.98 **	−0.69 **	−0.68 **		
AAC	−0.32	0.69 **	−0.51	0.83 **	0.78 **	0.73 **	0.98 **	−0.59 *	−0.73 **		
λmax	0.06	0.52	−0.10	0.85 **	0.54 *	0.83 **	−0.98 **	−0.30	−0.58 *		
Aλmax	−0.53	0.68 **	−0.70 **	0.79 **	0.69 **	0.68 **	0.98 **	−0.77 **	−0.86 **		
λmax/Aλmax	0.54 *	−0.60 *	0.68 **	−0.80 **	−0.51	−0.73 **	−0.98 **	0.80 **	0.93**		
Fb_3_	−0.52	0.67 **	−0.69 **	0.80 **	0.67 **	0.69 **	0.98 **	−0.77 **	−0.87 **		
	**Hardness**	**Toughness**	**Adhesion**	**Stickiness**	**Xylanase**	**Phosphorus**	**AAC**	**λ_max_**	**A_λmax_**	**λ_max_/** **A_λmax_**	**Fb_3_**
Hardness	1.00										
Toughness	0.65 *	1.00									
Adhesion	0.37	0.18	1.00								
Stickiness	0.38	0.66 *	0.15	1.00							
xylanase	0.38	0.24	−0.15	−0.02	1.00						
Phosphorus	0.67 **	0.23	0.12	0.07	0.09	1.00					
AAC	0.76 **	0.57 *	0.14	0.65 *	0.26	0.75 **	1.00				
λmax	0.32	0.49	−0.03	0.67 **	0.24	0.45	0.81 **	1.00			
Aλmax	0.79 **	0.54 *	0.31	0.58 *	0.12	0.81 **	0.96 **	0.73 **	1.00		
λmax/Aλmax	−0.63 *	−0.49	−0.38	−0.59 *	0.02	−0.73 **	−0.88**	−0.79 **	−0.96 **	1.00	
Fb_3_	0.77 **	0.54 *	0.30	0.59 *	0.11	0.81 **	0.96 **	0.75 **	1.00 **	−0.96 **	1.00

Correlation is significant at 5% (*) or 1% (**) by the method of *t*-test.

**Table 10:** Color difference of gelatinized paste and boiled rice soaked and boiled in 2 types of hard water or weakly acid hard water using Koshihikari of polished rice.

	**Cultivar/Water Types**	a*	b*	**ΔE (ab)**
Gelatinizied paste	Koshihikari ⋅ purified water	−2.94 ± 0.04 ^a^	−0.13 ± 0.30 ^c^	3.51 ± 0.06 ^b^
	Koshihikari ⋅ Evian (pH 7.2)	−2.88 ± 0.05 ^b^	0.59 ± 0.12 ^b^	3.47 ± 0.10 ^b^
	Koshihikari ⋅ Evian (pH 4.6)	−2.87 ± 0.02 ^b^	−0.22 ± 0.01 ^c^	3.39 ± 0.03 ^c^
	Koshihikari ⋅ Contrex (pH 7.2)	−2.94 ± 0.00 ^a^	1.05 ± 0.32 ^a^	3.74 ± 0.12 ^a^
	Koshihikari ⋅ Contrex (pH 4.6)	−2.77 ± 0.01 ^c^	−0.47 ± 0.16 ^d^	3.35 ± 0.01 ^c^
	**Cultivar/Water Types**	a*	b*	**ΔE (ab)**
Boiled rice	Koshihikari ⋅ purified water	−3.30 ± 0.04 ^a^	0.67 ± 0.01 ^c^	35.0 ± 0.72 ^b^
	Koshihikari ⋅ Evian (pH 7.2)	−3.43 ± 0.05 ^b^	1.58 ± 0.16 ^a^	36.79 ± 1.15 ^a^
	Koshihikari ⋅ Evian (pH 4.6)	−3.41 ± 0.07 ^b^	0.93 ± 0.01 ^b^	35.08 ± 0.47 ^b^
	Koshihikari ⋅ Contrex (pH 7.2)	−3.38 ± 0.03 ^a^	0.96 ± 0.13 ^b^	34.39 ± 0.22 ^c^
	Koshihikari ⋅ Contrex (pH 4.6)	−3.37 ± 0.04 ^a^	0.41 ± 0.16 ^d^	33.78 ± 0.16 ^d^

Different letters (^a–d^) indicate that samples soaked in different types of water significantly different. Values are shown as mean ± standard deviation.

## Data Availability

The original contributions presented in the study are included in the article/Supplementary Materials, further inquiries can be directed to the corresponding author.

## Supplementary Materials

The following are available online at https://www.mdpi.com/article/10.3390/foods13132094/s1, Figure S1: Sunshine hours in mid-August of 32 *Japonica* unpolished rice. Figure S2: Comparison of pasting properties of various starch samples and polished rice using purified water or Contrex with an RVA at 120 °C. Figure S3. Endo-xylanase activities of unpolished rice grains of various kinds of rice. Table S1: Alfa-amylase activities of unpolished rice soaking in two kinds of hard water (pH 7.2), weakly acid hard water (pH 4.6) and purified water and those of oligosaccharide contents. Table S2: Pasting properties with maintenance temperature 93 °C program of RVA for various kinds of polished rice using hard water, weakly acid hard water and purified water.

## Abbreviations

AAC: apparent amylose content; RS, resistant starch; SLC, super-long chains; DP, degree of polymerization; RVA, rapid visco analyser; SB, setback; BD, breakdown; Max. vis., maximum viscosity; Mini. vis., minimum viscosity; Pt, pasting temperature; Cons, consistency; Fin vis., final viscosity; Peak.t; Peak time.
