# Supplementary material for: Effects of Binding between Ca in Hard Water and Phosphorus in Amylopectin on the Qualities of Boiled Rice and Rice Noodle Prepared by Soaking and Boiling in Hard Water
Source: Foods. 2024 Jul 1;13(13):2094. doi: 10.3390/foods13132094 (PMC11241250; doi:10.3390/foods13132094)
Supplement: Supplementary file 1 [file foods-13-02094-s001.zip › foods-3060841-supplementary.pdf]

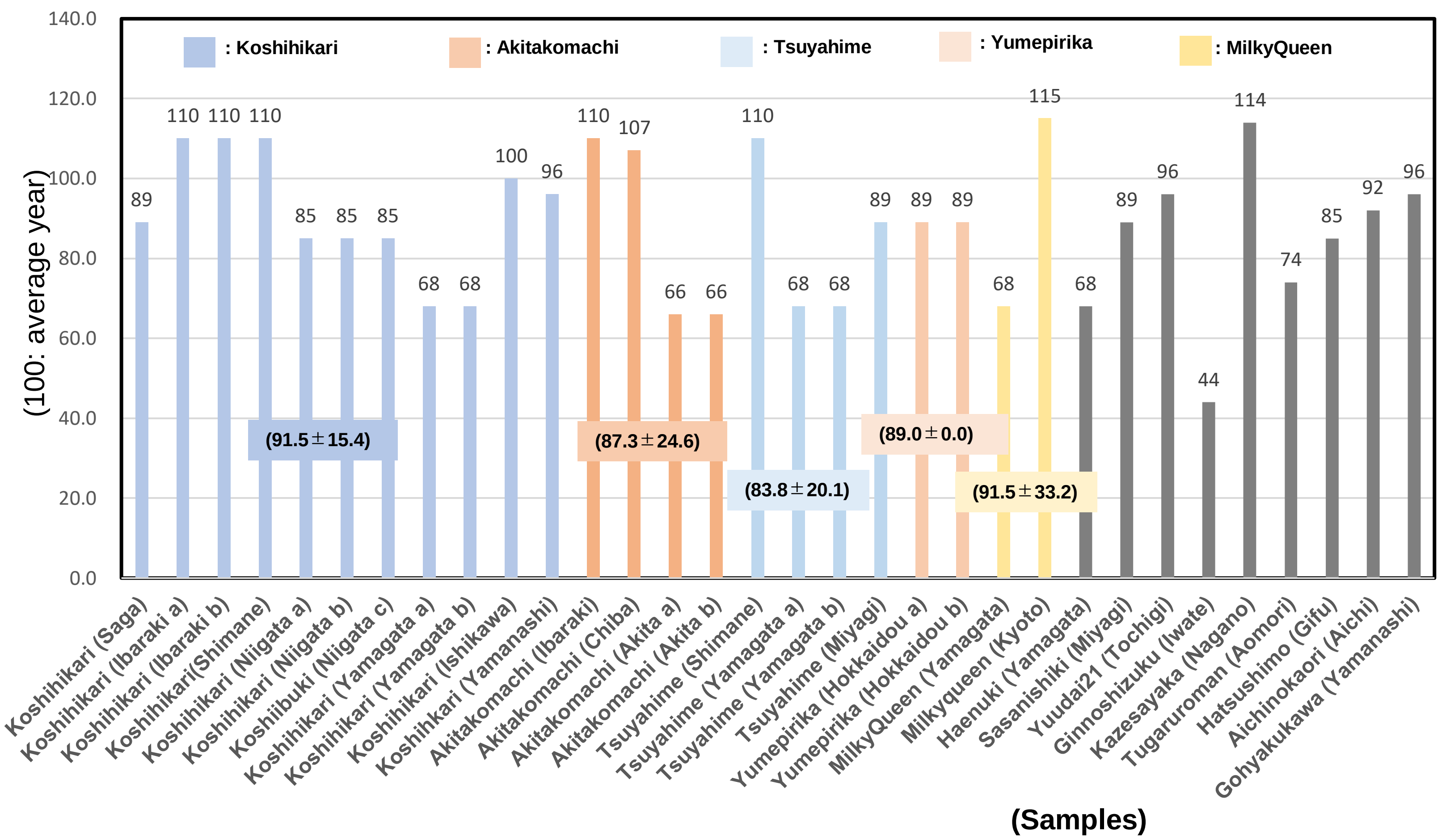

Values are shown as mean±standard deviation.

Fig S1. Sunshine hours in mid August of 32 unpolished ordinary *Japonica* rice in 2022.

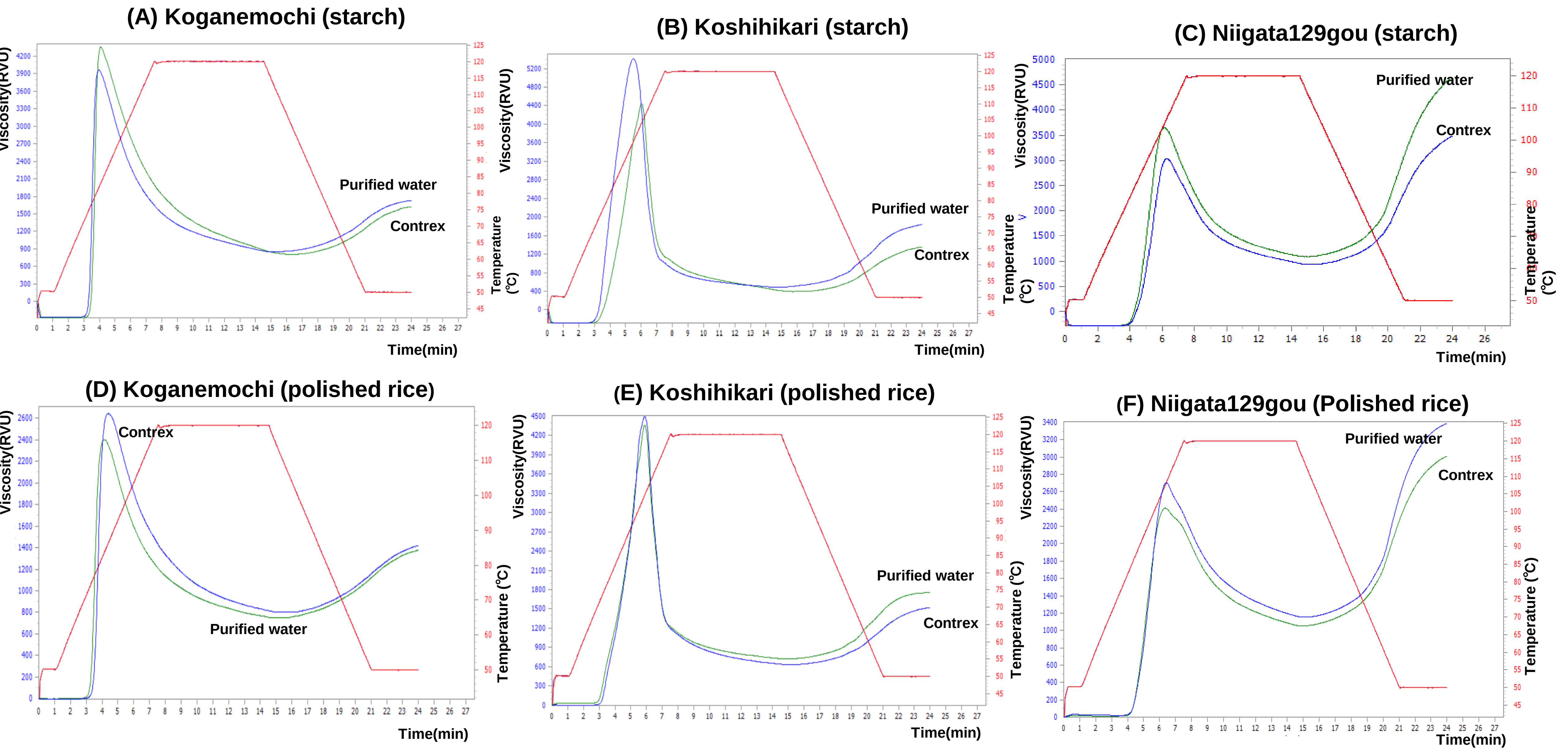

(A), (B), (C); Pasting properties of 3 kinds of rice starches using purified water or Contrex with an RVA at 120℃.
(D), (E), (F); Pasting properties of 3 kinds of polished rice using purified water or Contrex with an RVA at 120℃.

Figure S2. Comparison of pasting properties of various starch samples and polished rice using purified water or Contrex by RVA at 120℃.

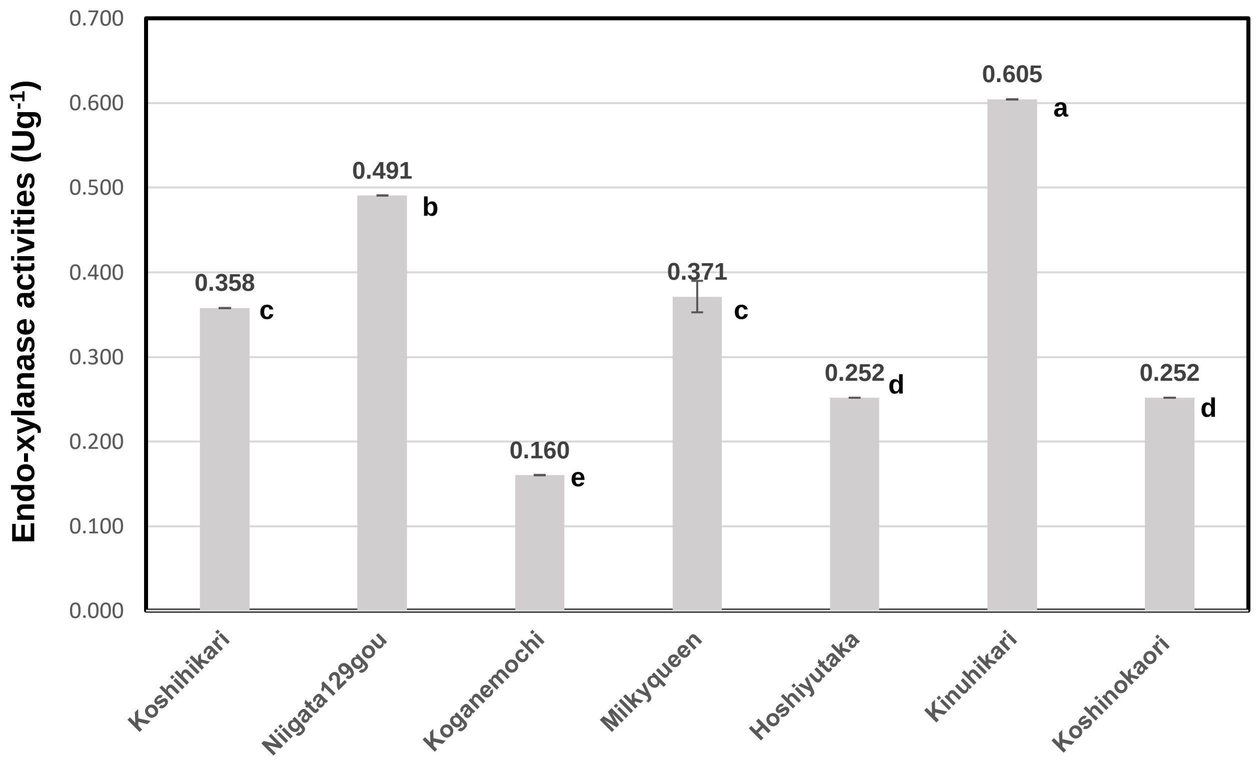

Different letter (a, b) indicate that unpolished rice grains samples are significantly different.
Correlation is significant at 1 % according to the method of Tukey's multiple comparison.

Fig S3. Endo-xylanase activities of unpolished rice grains of various kinds of rice in 2022.

	Kinds of water	α-Amylase (Ug^{-1})	D-Glucose Content (g/100g)
Koshihikari	purified water	0.034 ± 0.000 [a]	0.073 ± 0.002 [b]
	Evian	0.030 ± 0.000 [b]	0.048 ± 0.001 [c]
	Evian (pH 4.6)	0.030 ± 0.001 [b]	0.044 ± 0.002 [c]
	Contrex	0.033 ± 0.001 a	0.070 ± 0.003 [b]
	Contrex (pH4.6)	0.033 ± 0.001 [a]	0.152 ± 0.007 [a]
Kinuhikari	purified water	0.027 ± 0.002 [a]	0.075 ± 0.004 [b]
	Evian	0.026 ± 0.001 [a]	0.055 ± 0.000 [c]
	Evian (pH 4.6)	0.021 ± 0.001 [b]	0.045 ± 0.004 [d]
	Contrex	0.028 ± 0.001 [a]	0.072 ± 0.000 [b]
	Contrex (pH4.6)	0.029 ± 0.001 [a]	0.125 ± 0.004 [a]
Milkyqueen	purified water	0.058 ± 0.000 [a]	0.083 ± 0.003 [b]
	Evian	0.054 ± 0.003 [c]	0.054 ± 0.003 [d]
	Evian (pH 4.6)	0.053 ± 0.003 [c]	0.045 ± 0.001 [e]
	Contrex	0.056 ± 0.003 [b]	0.073 ± 0.003 [c]
	Contrex (pH4.6)	0.058 ± 0.003 [a]	0.126 ± 0.001 [a]

	Max.vis (cP)	Mini.vis (cP)	BD (cP)	Fin.vis (cP)	SB (cP)	Pt (℃)	Set/Cons	Max/Min	Max/Fin
Koshihikari・purified water	4653.0 ± 58.7 ^{c}	1137.0 ± 12.7 ^{b}	3516.5 ± 46.0 ^{c}	2234.5 ± 36.1 ^{b}	-2419.0 ± 22.6 ^{a}	60.5 ± 0.6 ^{b}	-2.2 ± 0.0 ^{a}	4.1 ± 0.0 ^{a}	2.1 ± 0.0 ^{a}
Koshihikari・Contrex	4953.0 ± 52.3 ^{a}	1249.5 ± 7.8 ^{a}	3703.5 ± 44.5 ^{a}	2375.0 ± 1.4 ^{a}	-2578.0 ± 53.7 ^{b}	63.5 ± 0.5 ^{a}	-2.3 ± 0.1 ^{a}	4.0 ± 0.0 ^{a}	2.1 ± 0.0 ^{a}
Koshihikari・Contrex (pH4.6)	4751.0 ± 26.9 ^{b}	1103.5 ± 6.4 ^{c}	3647,5 ± 20.5 ^{b}	2148.5 ± 9.2 ^{c}	-2602.5 ± 17.7 ^{c}	63.4 ± 0.7 ^{a}	-2.5 ± 0.0 ^{b}	4.3 ± 0.0 ^{a}	2.2 ± 0.0 ^{a}
Niigata129gou・purified water	2638.5 ± 21.9 ^{a}	2407.0 ± 12.7 ^{a}	231.5 ± 9.2 ^{c}	4165.5 ± 43.1 ^{a}	1527.0 ± 21.1 ^{a}	75.8 ± 0.5 ^{b}	0.9 ± 0.0 ^{a}	1.1 ± 0.0 ^{a}	0.6 ± 0.0 ^{a}
Niigata129gou・Contrex	1923.0 ± 29.7 ^{c}	1406.5 ± 14.8 ^{c}	516.5 ± 14.8 ^{a}	3084.5 ± 21.9 ^{c}	1161.5 ± 51.6 ^{c}	77.7 ± 0.2 ^{a}	0.7 ± 0.0 ^{a}	1.4 ± 0.2 ^{a}	0.6 ± 0.0 ^{a}
Niigata129gou・Contrex (pH4.6)	2275.5 ± 17.7 ^{b}	1948.5 ± 3.5 ^{b}	327.0 ± 21.2 ^{b}	3503.5 ± 27.6 ^{b}	1228.0 ± 45.3 ^{b}	74.4 ± 0.8 ^{b}	0.8 ± 0.0 ^{a}	1.2 ± 0.0 ^{a}	0.6 ± 0.0 ^{a}
Koganemochi・purified water	1991.5 ± 2.1 ^{c}	969.5 ± 4.9 ^{b}	1022.0 ± 7.1 ^{c}	1368.5 ± 2.1 ^{c}	-623.0 ± 4.2 ^{a}	50.3 ± 0.1 ^{a}	-1.6 ± 0.0 ^{b}	2.1 ± 0.0 ^{b}	1.5 ± 0.0 ^{a}
Koganemochi・Contrex	2220.0 ± 14.1 ^{b}	891.0 ± 9.9 ^{c}	1329.0 ± 24.0 ^{b}	1543.5 ± 0.7 ^{b}	'-676.5 ± 14.8 ^{b}	50.3 ± 0.2 ^{a}	-1.0 ± 0.0 ^{a}	2.5 ± 0.0 ^{a}	1.4 ± 0.0 ^{a}
Koganemochi・Contrex(pH4.6)	2792.5 ± 12.0 ^{a}	1083.5 ± 0.7 ^{a}	1709.0 ± 11.3 ^{a}	1702.0 ± 0.0 ^{a}	'-1090.5 ± 12.0 ^{c}	50.3± 0.0 ^{a}	-1.8 ± 0.0 ^{b}	2.6 ± 0.0 ^{a}	1.6 ± 0.0 ^{a}
Milkyqueen・purified water	4488.5 ± 44.5 ^{c}	978.0 ± 6.4 ^{a}	3510.0 ± 38.2 ^{c}	1671.0 ± 25.5 ^{a}	'-2817.5 ± 19.1 ^{a}	61.3 ± 2.1 ^{a}	-4.1 ± 0.1 ^{a}	4.6 ± 0.0 ^{b}	2.7 ± 0.0 ^{a}
Milkyqueen・Contrex	4742.5 ± 6.4 ^{a}	934.0 ± 22.6 ^{b}	3808.5 ± 29.0 ^{a}	1622.5 ± 2.1 ^{b}	-3120.0 ± 8.5 ^{c}	59.8 ± 1.1 ^{b}	-4.5 ± 0.1 ^{b}	5.1 ± 0.1 ^{a}	2.9 ± 0.0 ^{a}
Milkyqueen・Contrex (pH4.6)	4582.0 ± 14.1 ^{b}	965.5 ± 6.4 ^{a}	3616.5 ± 7.8 ^{b}	1616.0 ± 1.4 ^{b}	'-2966.0 ± 12.7 ^{b}	59.6 ± 1.6 ^{b}	'-4.6 ± 0.1 ^{b}	4.7 ± 0.0 ^{b}	2.8 ± 0.0 ^{a}
Hoshiyutaka・purified water	3673.5 ± 12.0 ^{a}	1471.0 ± 8.5 ^{a}	2202.5 ± 3.5 ^{a}	4581.5 ±12.0 ^{a}	908.0 ± 0.0 ^{a}	59.1 ± 0.7 ^{a}	0.3 ± 0.0 ^{a}	2.5 ± 0.2 ^{b}	0.8 ± 0.0 ^{b}
Hoshiyutaka・Contrex	3164.5 ± 9.2 ^{c}	950.5 ± 3.5 ^{c}	2214.0 ± 5.7 ^{a}	2956.5 ± 3.5 ^{c}	-208.0 ± 5.7 ^{c}	59.8 ± 1.2 ^{a}	-0.1 ± 0.0 ^{b}	3.3 ± 0.0 ^{a}	1.1 ± 0.0 ^{a}
Hoshiyutaka・Contrex(pH4.6)	3284.5 ± 12.0 ^{b}	1389.5 ± 2.1 ^{b}	1895.0 ± 14.1 ^{b}	3754.5 ± 4.9 ^{b}	470.0 ± 7.1 ^{b}	58.2 ± 1.3 ^{b}	0.2 ± 0.0 ^{a}	2.4 ± 0.0 ^{b}	0.9 ± 0.0 ^{b}
Kinuhikari・purified water	5087.0 ± 9.9 ^{c}	1304.0 ± 8.5 ^{b}	3783.0 ± 1.4 ^{c}	2342.5 ± 2.1 ^{b}	-2744.5 ± 7.8 ^{b}	65.8 ± 0.6 ^{b}	-2.6 ± 0.0 ^{a}	3.9 ± 0.0 ^{b}	2.2 ± 0.0 ^{a}
Kinuhikari・Contrex	5350.5 ± 43.1 ^{a}	1508.0 ± 42.4 ^{a}	3842.5 ± 0.7 ^{b}	2639.0 ± 26.9 ^{a}	-2711.5 ± 16.3 ^{a}	62.0 ± 0.9 ^{c}	-2.4 ± 0.0 ^{a}	3.5 ± 0.1 ^{c}	2.0 ± 0.0 ^{a}
Kinuhikari・Contrex (pH4.6)	5185.5 ± 17.7 ^{b}	1217.5 ± 3.5 ^{c}	3968.0 ± 21.2 ^{a}	2211.5 ± 4.9 ^{c}	-2974.0 ± 22.6 ^{c}	67.6 ± 0.8 ^{a}	-3.0 ± 0.0 ^{b}	4.3 ± 0.0 ^{a}	2.3 ± 0.0 ^{a}
Koshinokaori・purified water	3498.0 ± 67.9 ^{a}	1208.5 ± 34.6 ^{b}	2289.5 ± 33.2 ^{a}	3425.0 ± 25.5 ^{a}	'-73.0 ± 42.4 ^{b}	58.8 ± 1.9 ^{b}	0.0 ± 0.0 ^{a}	2.9 ± 0.0 ^{a}	1.0 ± 0.0 ^{a}
Koshinokaori・Contrex	3317.5 ±17.7 ^{b}	1311.0 ± 11.3 ^{a}	2006.5 ± 29.0 ^{b}	3364.0 ± 9.9 ^{b}	'46.5 ± 27.6 ^{a}	60.0 ± 1.4 ^{b}	0.0 ± 0.0 ^{a}	2.5 ± 0.0 ^{b}	1.0 ± 0.0 ^{a}
Koshinokaori・Contrex (pH4.6)	3434.5 ± 7.82 ^{a}	1200.0 ± 2.8 ^{b}	2234.5 ± 4.9 ^{a}	3235.5 ± 16.3 ^{c}	-199.0 ± 8.5 ^{c}	63.9 ± 1.8 ^{a}	-0.1 ± 0.0 ^{a}	2.9 ± 0.0 ^{a}	1.1 ± 0.0 ^{a}
